# A novel function of cIAP1 as a mediator of CHIP-driven eIF4E regulation

**DOI:** 10.1038/s41598-017-10358-2

**Published:** 2017-08-29

**Authors:** Tae Woong Seo, Ji Sun Lee, Ye Na Choi, Dar Heum Jeong, Sun Kyung Lee, Soon Ji Yoo

**Affiliations:** 1 Department of Life and Nanopharmaceutical Sciences, Kyung Hee University, Seoul, 02447 Korea; 2 Department of Biology, Kyung Hee University, Seoul, 02447 Korea

## Abstract

eIF4E is an initiator protein in cap-dependent translation. Its overexpression is linked to tumorigenesis in various human cancers, suggesting that the levels of eIF4E must be under tight control in normal cells. Although several eIF4E regulatory mechanisms have been demonstrated, the intracellular mechanisms controlling eIF4E protein levels remain poorly understood. Here, we report that eIF4E is efficiently regulated by dual mechanisms, both involving human inhibitor of apoptosis family protein cIAP1. cIAP1 itself ubiquitinates eIF4E as an E3 ligase, and interestingly, cIAP1 also functions as a mediator to present eIF4E to another E3 ligase, CHIP. This collaborative activity of cIAP1 and CHIP directs eIF4E toward degradation, controlling its levels and suppressing tumorigenesis. Our results provide the first evidence for a mediator function of cIAP1 and collaborative activity of cIAP1 and CHIP, suggesting that maintaining balanced levels of these E3 ligases might be beneficial for normal cell growth.

## Introduction

Inhibitor of apoptosis proteins (IAPs) are evolutionarily conserved from viruses to mammals. Human IAPs contain 1 to 3 baculoviral IAP repeat (BIR) domains, and several of them also contain really interesting new gene (RING) domains^1^. Originally, IAPs were identified as negative regulators of apoptosis acting through direct binding of their BIR domains to caspases^2^. In recent years, RING domains have been shown to have E3 ligase activity^3^, and this function is present in IAPs cIAP1^4–6^, cIAP2^6, 7^, and XIAP^8, 9^. This suggests that IAPs participate not only in regulation of apoptosis but also in a wide range of cellular events as E3 ligases.

Cap-dependent translation begins with the binding of eIF4E to the 5ʹcap of an mRNA and subsequent formation of the eIF4F complex. Therefore, eIF4E availability is crucial for initiation of cap-dependent translation. The eIF4E-binding protein (4EBP) is an inhibitor of eIF4E that is phosphorylated in response to growth-stimulating signals. Unphosphorylated 4EBP sequesters eIF4E from binding to the 5ʹcap structure, while phosphorylated 4EBP cannot bind to eIF4E and thus allows eIF4E to bind the 5ʹcap structure^10^. The control of eIF4E availability by 4EBP is effective when intracellular levels of eIF4E are equivalent to levels of 4EBP. However, if eIF4E levels somehow exceed 4EBP in dynamic cellular environments and thus cannot be controlled by 4EBP, uncontrolled eIF4E might increase global protein synthesis and have harmful consequences. In support of this hypothesis, ectopic expression of eIF4E is sufficient to transform several normal cell lines^11^, and eIF4E is overexpressed up to 10-fold in many human cancers^12^. Especially in breast cancers, high levels of eIF4E are associated with increased cancer recurrence and poor survival^13, 14^. Furthermore, a recent report showed that reduction of eIF4E expression by 50% is compatible with normal development and eIF4E exceeding the levels for normal development can drive tumorigenesis^15^. These studies collectively suggest that the levels of eIF4E are closely linked to maltransformation of cells, and that there must therefore be tight regulatory mechanisms controlling the levels of eIF4E protein.

C-terminus of Hsc-70 interacting protein (CHIP) is a U-box type E3 ubiquitin ligase. CHIP was originally identified as a quality-control E3 ligase that ubiquitinates misfolded or abnormal proteins presented by chaperones^16^. However, CHIP has also recently been shown to ubiquitinate specific target proteins for proteasomal degradation^17–19^. Interestingly, several reports showed that CHIP is able to regulate certain target proteins in a chaperone-independent manner^20–23^, suggesting that the molecular mechanisms of CHIP-driven ubiquitination may be diverse and dependent on the target protein. Although ubiquitination of eIF4E by CHIP has been shown in human cell lines^24^, the precise molecular mechanism by which CHIP regulates eIF4E has yet to be elucidated.

We have previously reported that, in *Drosophila*, deIF4E is ubiquitinated by *Drosophila* inhibitor of apoptosis protein (Diap1) for proteasomal degradation^25^. In the present study, we sought to investigate whether eIF4E is regulated by any of the human IAPs, which led to the discovery of an interesting mechanism of eIF4E regulation. Here, we describe this collaborative role of cIAP1 and CHIP in eIF4E regulation, and a novel function of cIAP1 as a mediator of CHIP-driven regulation of eIF4E.

## Results

### Human IAP cIAP1 specifically interacts with eIF4E

**Figure 1:**
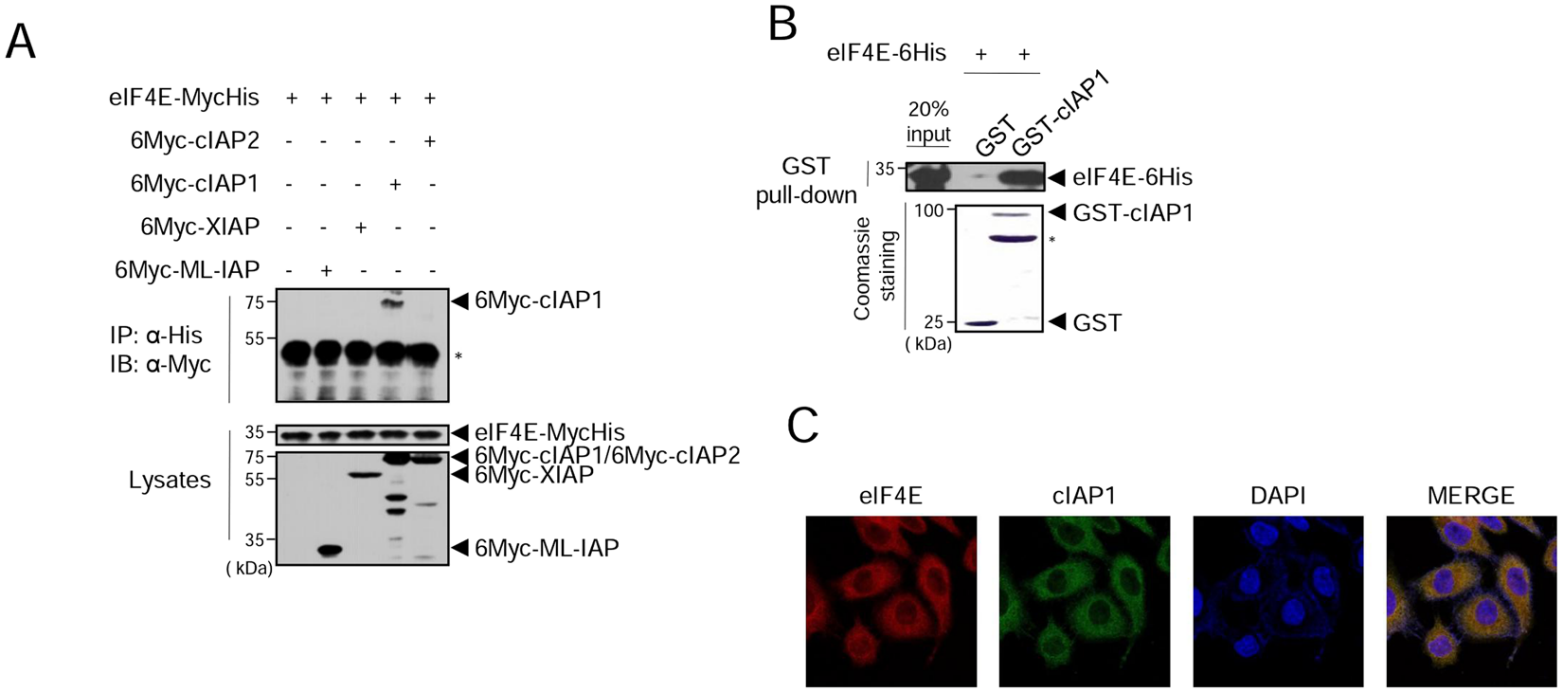
Among human IAPs, cIAP1 specifically interacts with eIF4E. (**A**) HEK 293 cells were co-transfected with 6Myc-ML-IAP, 6Myc-XIAP, 6Myc-cIAP1, 6Myc-cIAP2, or eIF4E-MycHis. After 24 h, whole cell lysates (WCL) were co-immunoprecipitated with anti-His antibody analyzed by western blot (WB) with anti-Myc antibody (top). Expression of IAPs and eIF4E was determined by WB using anti- His or anti-Myc (bottom) antibodies. *IgG heavy chain. (**B**) eIF4E-6His protein was incubated with GST or GST-cIAP1 proteins on glutathione beads. Precipitated eIF4E-6His protein was subjected to WB with anti-His antibody. *Non-specific or GST-cIAP1 fragment band. (**C**) HeLa cells were immunostained with anti-eIF4E (red) and anti-cIAP1 (green) antibodies. DAPI (blue) was used to counterstain nuclear DNA. WB images are cropped from the original blots shown in supplementary information.

Given that both IAPs and eIF4E are evolutionally conserved^1, 25^, we hypothesized that eIF4E and IAPs might interact in human cells. We co-expressed Myc- and His-tagged eIF4E protein (eIF4E-MycHis) and one of four human IAPs (6Myc-cIAP1, 6Myc-cIAP2, 6Myc-XIAP, 6Myc-ML-IAP) in HEK 293 cells and examined their interaction using co-immunoprecipitation (co-IP) experiments. The results showed that only cIAP1 interacted with eIF4E-MycHis (Fig. 1A). In a GST pull-down assay, western blot (WB) analysis showed that eIF4E was captured by GST-cIAP1 beads but not by control GST beads (Fig. 1B), indicating that cIAP1 directly interacted with eIF4E. When we examined their localization in HeLa cells, eIF4E and cIAP1 both were mainly observed in the cytoplasm (Figs 1C and S1). These results demonstrated that, among human IAPs, cIAP1 specifically interacts with eIF4E in human cells, implying that the interaction between eIF4E and IAPs is evolutionarily conserved from fly to human.

### The levels of eIF4E protein are regulated by cIAP1

**Figure 2:**
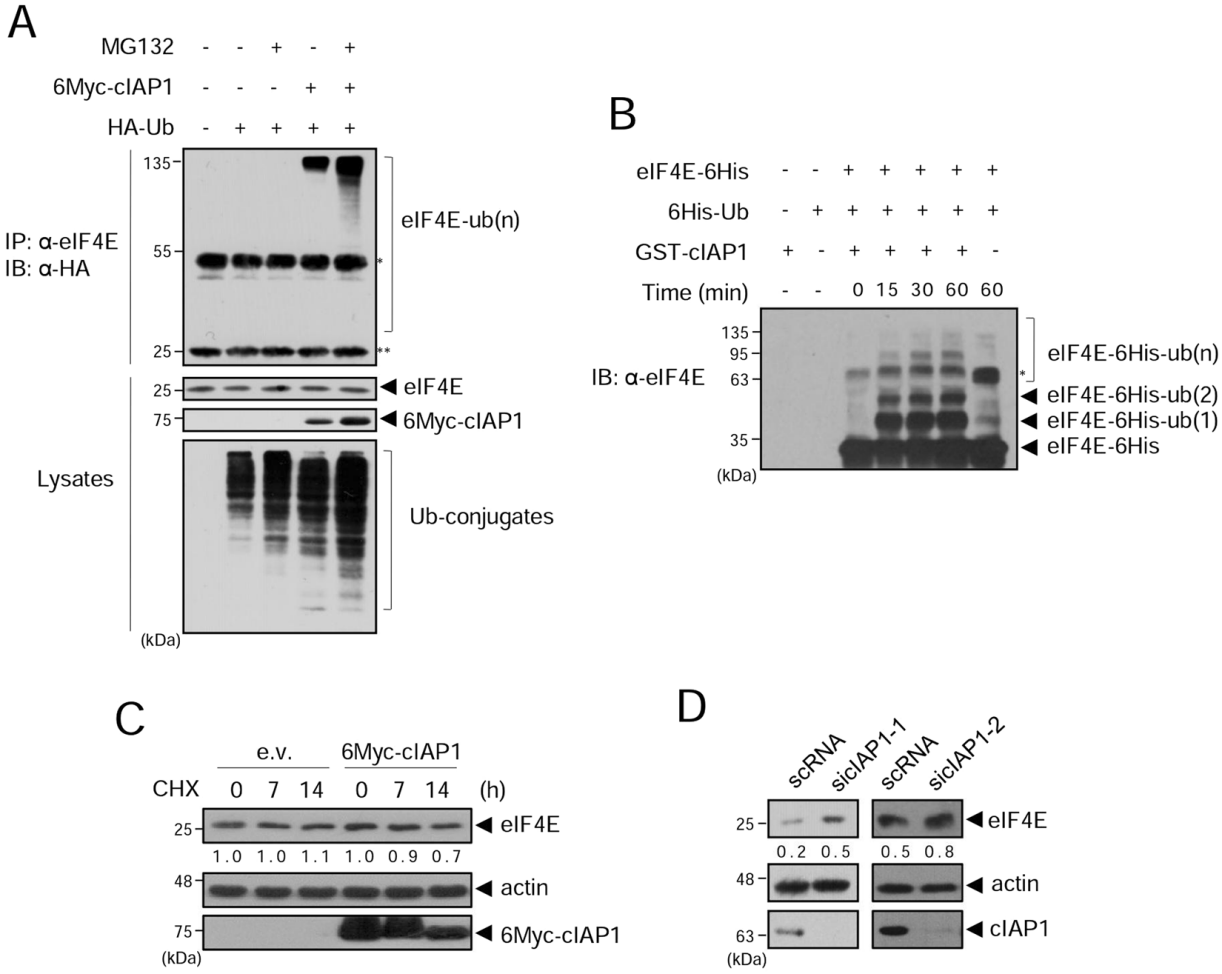
The ubiquitination and levels of eIF4E protein are regulated by cIAP1. (**A**) HEK 293 T cells were co-transfected with 6Myc-cIAP1 and HA-ubiquitin. After 24 h, cells were treated with 20 μM of MG132 for 6 h, and WCL were co-immunoprecipitated with anti-eIF4E antibody and analyzed by WB with anti-HA antibody. *IgG heavy chain, **IgG light chain. (**B**) Purified eIF4E-6His and GST-cIAP1 proteins were subjected to the *in vitro* ubiquitination assay. After incubation, samples were analyzed by WB with anti-eIF4E antibody. *Non-specific background band after 1 h incubation without GST-cIAP1. (**C**) HeLa cells were transfected with empty vector (e.v.) or 6Myc-cIAP1. After 48 h, cells were treated with 200 μg/ml cycloheximide (CHX) and harvested at the indicated times. WCL were analyzed by WB with anti-eIF4E and anti-Myc antibodies. Actin was used as a quantitative loading control. The numbers indicate relative eIF4E expression to actin within the indicated vector-expressing cells. (**D**) HeLa cells were transfected with control scRNA (scRNA) or cIAP1-targeted siRNAs (sicIAP1-1, sicIAP1-2). WCL were analyzed by WB with anti-eIF4E, anti-cIAP1, and anti-actin antibodies at two days after transfection. The numbers indicate expression of eIF4E relative to actin. WB images are cropped from the original blots shown in supplementary information.

To examine whether cIAP1 regulates eIF4E in a proteasome-dependent manner, we expressed 6Myc-cIAP1 in HEK 293 T cells in the presence or absence of proteasome inhibitor MG132, and performed an *in vivo* ubiquitination assay. We found that endogenous eIF4E was ubiquitinated by cIAP1, and ubiquitinated eIF4E accumulated upon MG132 treatment (Fig. 2A). We carried out an *in vitro* ubiquitination assay with purified eIF4E-6His and GST-cIAP1. Mono-, di-, and polyubiquitinated eIF4E proteins were detected in the presence of GST-cIAP1, and the amount of polyubiquitinated eIF4E gradually increased over time (Figs 2B and S2), indicating that eIF4E is specifically ubiquitinated by cIAP1. Next, we analyzed the half-life of eIF4E after blocking translation using cycloheximide (CHX) treatment. As shown in Fig. 2C, the half-life of eIF4E in HeLa cells overexpressing 6Myc-cIAP1 was reduced compared to that in control cells. Interestingly, eIF4E showed a similar half-life also in HeLa cells overexpressing 6Myc-cIAP1 RING mutant (RM, H588A/C592A), an E3 inactive mutant of cIAP1 (Fig. S3), suggesting cIAP1 may have another function in regulation of eIF4E more than an E3 ligase. Finally, we examined whether eIF4E protein was stabilized when cIAP1 was depleted. The levels of eIF4E were elevated in cIAP1-depleted cells compared to control (Fig. 2D). Altogether, these results demonstrate that cIAP1 regulates the steady-state levels of eIF4E protein.

### The levels of eIF4E are regulated by CHIP in a molecular chaperone-independent manner

**Figure 3:**
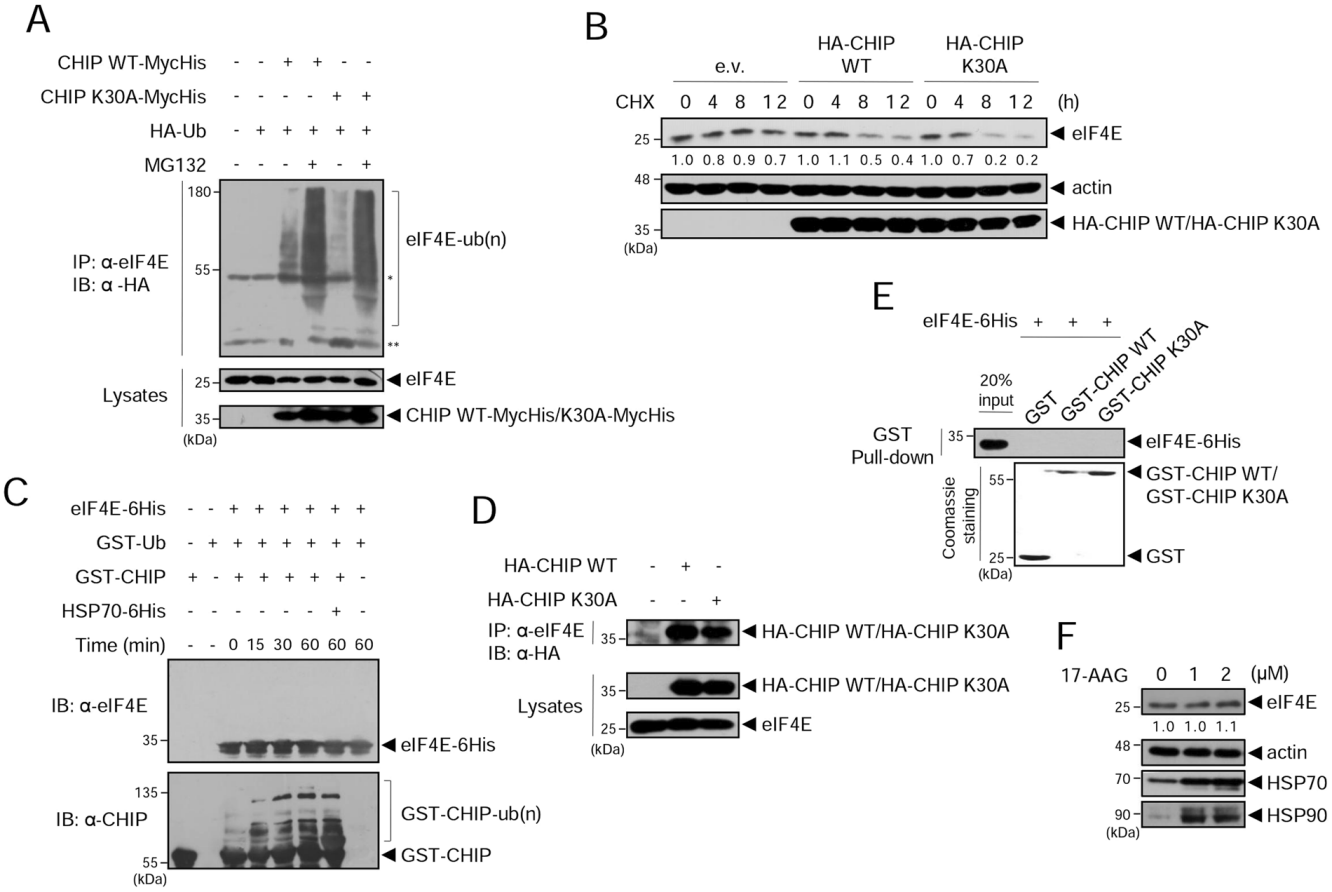
eIF4E is regulated by CHIP but not via direct binding. (**A**) HEK 293 T cells were co-transfected with HA-ubiquitin and CHIP WT-MycHis or CHIP K30A-MycHis. After 24 h, cells were treated with 20 μM MG132 for 6 h, and WCL were co-immunoprecipitated with anti-eIF4E antibody, followed by WB with anti-HA antibody. *IgG heavy chain, **IgG light chain. (**B**) HeLa cells were transfected with HA-CHIP WT, HA-CHIP K30A, or empty vector (e.v.). After 48 h, cells were treated with 200 μg/ml CHX and harvested at the indicated times. WCL were analyzed by WB with anti-eIF4E and anti-HA antibodies. The numbers indicate relative eIF4E expression to actin within the indicated vector-expressing cells. (**C**) The in vitro ubiquitination assay using purified eIF4E-6His, GST-CHIP, and Hsp70-6His was analyzed by WB with anti-eIF4E antibody (top) and with anti-CHIP antibody (bottom). (**D**) HEK 293 cells were transfected with HA-CHIP WT or HA-CHIP K30A. After 24 h, WCL were co-immunoprecipitated with anti-eIF4E antibody and WB with anti-HA antibody. (**E**) eIF4E-6His protein was incubated with GST, GST-CHIP WT, or GST-CHIP K30A proteins on glutathione beads. Precipitated eIF4E-6His protein was subjected to SDS-PAGE and WB with anti-His antibody. (**F**) MDA-MB231 cells were treated with 17-(Allylamino)geldanamycin (17-AAG) for 24 h and WCL were analyzed by WB with the indicated antibodies. The numbers indicate relative eIF4E expression to actin. WB images are cropped from the original blots shown in supplementary information.

CHIP has been previously reported to ubiquitinate eIF4E^24^. It is not surprising that a crucial protein such as eIF4E would be regulated by multiple E3 ligases, so we were interested in how eIF4E is differently regulated by two E3 ligases, cIAP1 and CHIP. CHIP has recently been shown to regulate specific target proteins in a molecular chaperone-independent manner^17–19^. Therefore, we investigated whether a molecular chaperone is required for eIF4E ubiquitination by CHIP. We compared eIF4E ubiquitination by CHIP WT and CHIP K30A which is a mutant incapable of binding to chaperones^26^. Co-IP showed that CHIP K30A ubiquitinated eIF4E to a similar extent as CHIP WT did (Fig. 3A). As a result, the half-life of eIF4E was reduced in cells overexpressing CHIP K30A as much as it was in cells overexpressing CHIP WT (Fig. 3B), while the E3 inactive mutant CHIP ∆U-box did not affect the half-life of eIF4E (Fig. S4). These results collectively suggest that the regulation of eIF4E by CHIP may not require a molecular chaperone. As a parallel experiment, we performed an *in vitro* ubiquitination assay. Interestingly, eIF4E was not ubiquitinated by purified CHIP (Fig. 3C, top). We examined the auto-ubiquitination of GST-CHIP by re-blotting the same gel with anti-CHIP antibody, to verify that purified GST-CHIP WT and other components in the reaction mixture were active. GST-CHIP was well auto-ubiquitinated (Fig. 3C, bottom), demonstrating that GST-CHIP and the other components were active. We added Hsp70 to the assay to determine whether Hsp70 might support eIF4E ubiquitination by CHIP, but no ubiquitinated eIF4E was observed (Fig. 3C, top). Next, we tested the interaction of eIF4E with CHIP *in vivo* and *in vitro*. Co-IP results showed that endogenous eIF4E interacted with CHIP WT and also with CHIP K30A (Fig. 3D). However, in an *in vitro* GST pull-down assay, eIF4E did not bind to either GST-CHIP WT or K30A beads (Fig. 3E), indicating that eIF4E does not directly interact with CHIP. This discrepancy between *in vivo* and *in vitro* results raised the possibility that there might be a mediator that directs eIF4E to CHIP. A reasonable candidate as a mediator would be Hsp70; however, CHIP K30A, which is unable to interact with Hsp70, also regulated eIF4E (Fig. 3A,B), and Hsp70 did not support eIF4E ubiquitination by CHIP *in vitro* (Fig. 3C). Finally, to confirm that CHIP-driven eIF4E ubiquitination is chaperone-independent, we treated MDA-MB231 cells with 17-(Allylamino)geldanamycin (17-AAG), a potent antitumor drug that inhibits Hsp90, which causes destabilization of its effector proteins via Hsp70 and CHIP^27^. 17-AAG treatment has been shown to increase Hsp90 and Hsp70 in MDA-MB231 cells^28, 29^, indicating that 17-AAG actually worked. The eIF4E should have been decreased on 17-AAG treatment if it was regulated in chaperone dependent manner. However, eIF4E levels were not changed while Hsp90 and Hsp70 were increased (Fig. 3F), consistent with chaperone-independency in CHIP-driven eIF4E regulation.

### cIAP1 and CHIP physically interact

**Figure 4:**
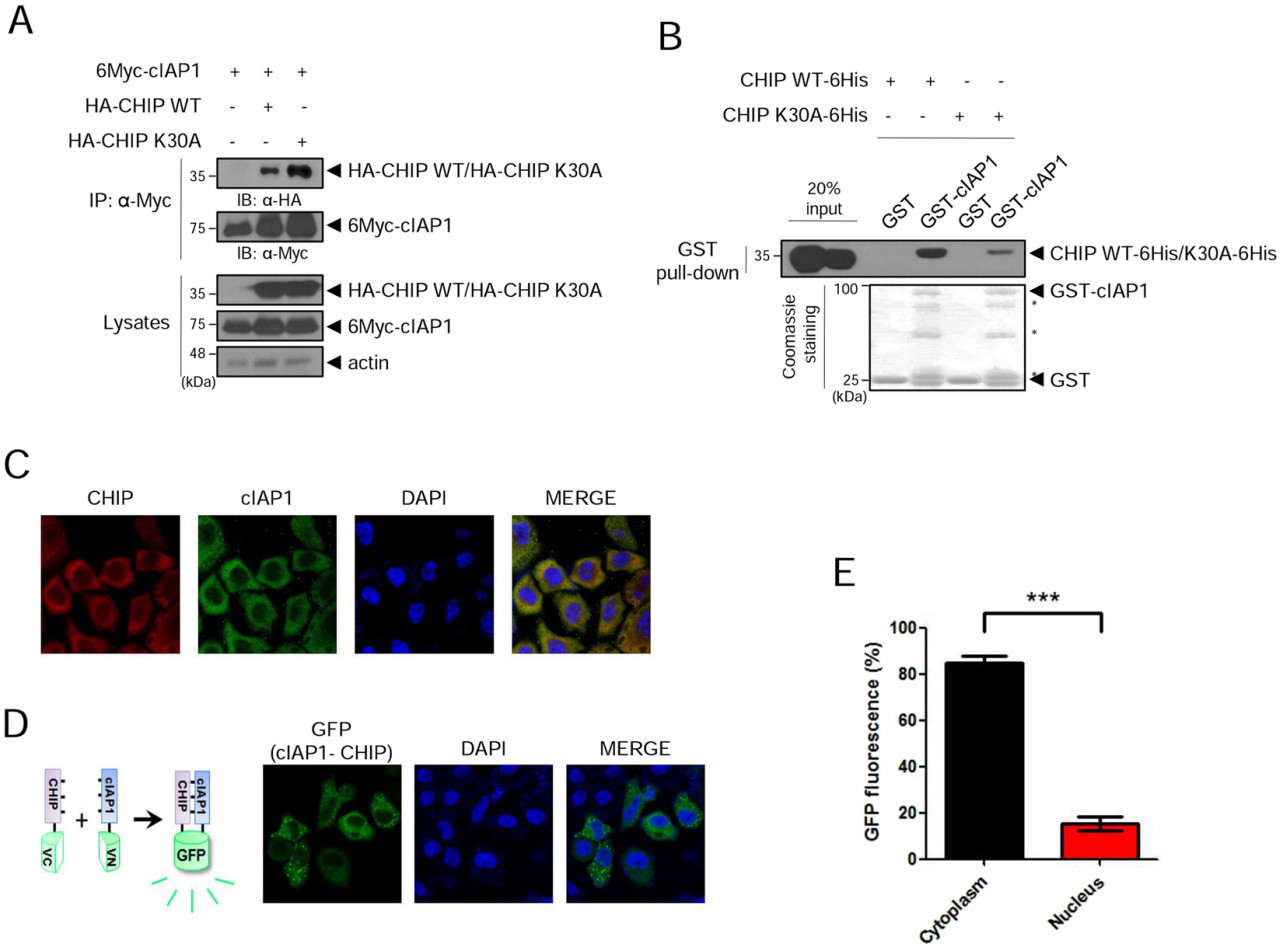
cIAP1 and CHIP interact in vitro and *in vivo*. (**A**) HEK 293 cells were co-transfected with 6Myc-cIAP1 and HA-CHIP WT or HA-CHIP K30A. After 24 h, WCL were co-immunoprecipitated with anti-Myc antibody and analyzed by WB with anti-HA antibody. (**B**) Purified GST and GST-cIAP1 proteins on glutathione beads were incubated with purified CHIP WT-6His or CHIP K30A-6His proteins. After incubation, the beads were analyzed by WB using anti-His antibody. *GST-cIAP1 fragments or non-specific bands. (**C**) HeLa cells were immunostained with anti-cIAP1 antibody (green) and anti-CHIP antibody (red). DAPI (blue) was used to counterstain nuclear DNA. (**D**) Hela cells were co-transfected with cIAP1-VN and CHIP-VC for BiFC assay. After fixation and permeabilization, cells were stained with DAPI to visualize the nucleus. The interaction of the two proteins was visualized by GFP fluorescence. (**E)** Quantification of GFP fluorescence per cell in Hela cells using Image J program. Values represent means ± s.e.m from triplicate independent experiments (***p < 0.0001, unpaired t-test). WB images are cropped from the original blots shown in supplementary information.

CHIP has been shown to interact with RING-type E3 ligases including Mdm2 and Parkin and to regulate their activities^30–32^. Because the RING-type E3 ligase cIAP1 regulated eIF4E (Fig. 2), we hypothesized that CHIP and cIAP1 might interact to regulate eIF4E. Co-IP showed that cIAP1 bound to CHIP WT and also to CHIP K30A (Fig. 4A). In a GST pull-down assay, CHIP WT-6His and CHIP K30A-6His bound to GST-cIAP1 beads but not GST beads (Fig. 4B), indicating that the interaction between cIAP1 and CHIP was a direct physical interaction. Next, we examined the localization of endogenous cIAP1 and CHIP by immunocytochemistry in HeLa cells. Both proteins were mainly localized in the cytoplasm (Figs 4C and S5). We further investigated the direct interaction of cIAP1 with CHIP using the bimolecular fluorescence complementation (BiFC) assay, in which green fluorescent signal is emitted if the proteins fused to each half of the GFP (N-terminal or C-terminal fragment) interact *in vivo* ^33^. We generated the fusion proteins—cIAP1 fused to the N-terminal half (cIAP1-VN) and CHIP fused to the C-terminal half (CHIP-VC) of the Venus fluorescent protein (Fig. 4D, left)—and co-expressed them in Cos7 cells. Bright green fluorescence was almost exclusively observed in the cytoplasm (Figs 4D, right, E and S6), indicating that cIAP1 and CHIP directly interact in the cytoplasm.

### cIAP1 mediates the interaction of eIF4E with CHIP

**Figure 5:**
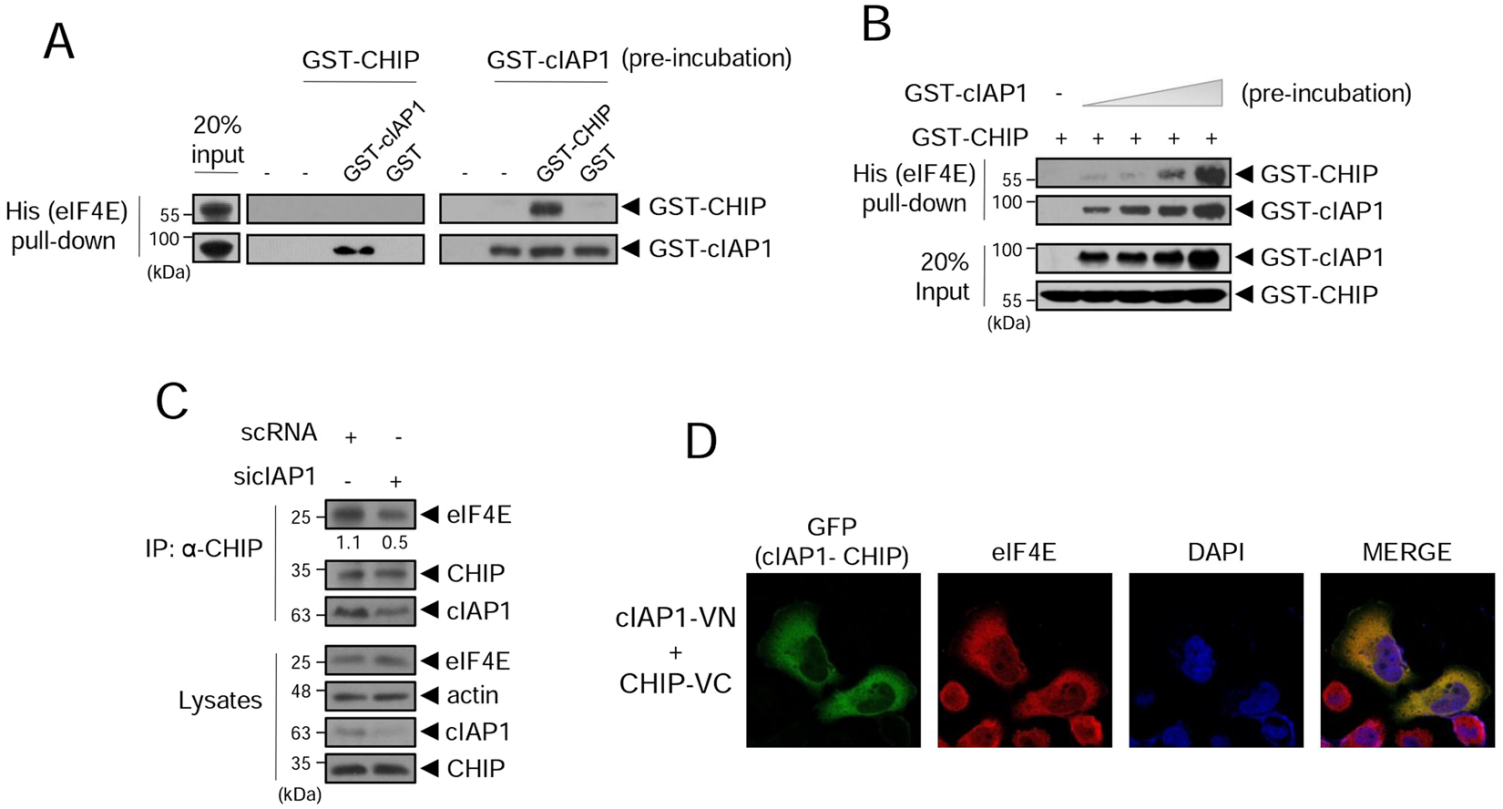
Interaction of eIF4E with CHIP is dependent on cIAP1. (**A**) Purified GST, GST-CHIP, GST-cIAP1, and eIF4E-6His proteins were used in the His pull-down assay. After incubation, the beads were analyzed by WB using anti-GST antibody. (**B**) His pull-down assay with increased concentration of GST-cIAP1 proteins. Complexes of eIF4E-6His, GST-cIAP1, and GST-CHIP were analyzed by SDS-PAGE and WB using anti-GST antibody. (**C**) HeLa cells were transfected with scRNA or sicIAP1. After 24 h transfection, WCL were immunoprecipitated with anti-CHIP antibody, and analyzed by WB with the indicated antibodies. The numbers indicate levels of co-immunoprecipitated eIF4E relative to immunoprecipitated CHIP. (**D**) HeLa cells were co-transfected with cIAP1-VN and CHIP-VC. After 24 h transfection, cells were immunostained with anti-eIF4E antibody (red) and DAPI to visualize the nucleus (blue). GFP fluorescence indicating an interaction between cIAP1 and CHIP was merged with the endogenous eIF4E staining (red) in the cytoplasm. WB images are cropped from the original blots shown in supplementary information.

Because cIAP1 interacts directly with both CHIP (Fig. 4) and eIF4E (Fig. 1), we reasoned that cIAP1 might mediate the interaction of eIF4E with CHIP. To test this possibility, we designed an *in vitro* His-tag pull-down assay. First, eIF4E-6His beads were pre-incubated with GST-CHIP or GST-cIAP1, then the beads were incubated with either the converse GST-tagged protein or GST alone. GST-cIAP1 was not detected on the beads that had been pre-incubated with GST-CHIP (Fig. 5A, left). However, GST-CHIP and GST-cIAP1 were both detected on beads pre-incubated with GST-cIAP1 (Fig. 5A, right), indicating that eIF4E-6His, GST-cIAP1, and GST-CHIP are able to form a complex when the beads are pre-incubated with GST-cIAP1. Binding of GST-CHIP to eIF4E-6His beads increased in proportion to the amount of GST-cIAP1 used in the pre-incubation (Fig. 5B). Co-IP with anti-CHIP antibody showed that co-immunoprecipitated eIF4E was decreased less than 50% in cIAP1-depleted cells (sicIAP1) compared to that in control cells (scRNA) (Fig. 5C). Additionally, we confirmed that cIAP1, CHIP, and eIF4E were mainly localized in the cytoplasm using the BiFC assay and eIF4E immunostaining (Figs 5D and S7). Altogether, these results demonstrate that cIAP1 mediated the interaction of eIF4E with CHIP, thereby allowing the formation of an eIF4E, cIAP1, and CHIP complex in the cytoplasm.

### The BIR1 domain of cIAP1 is responsible for eIF4E binding while BIR3 is responsible for CHIP binding

**Figure 6:**
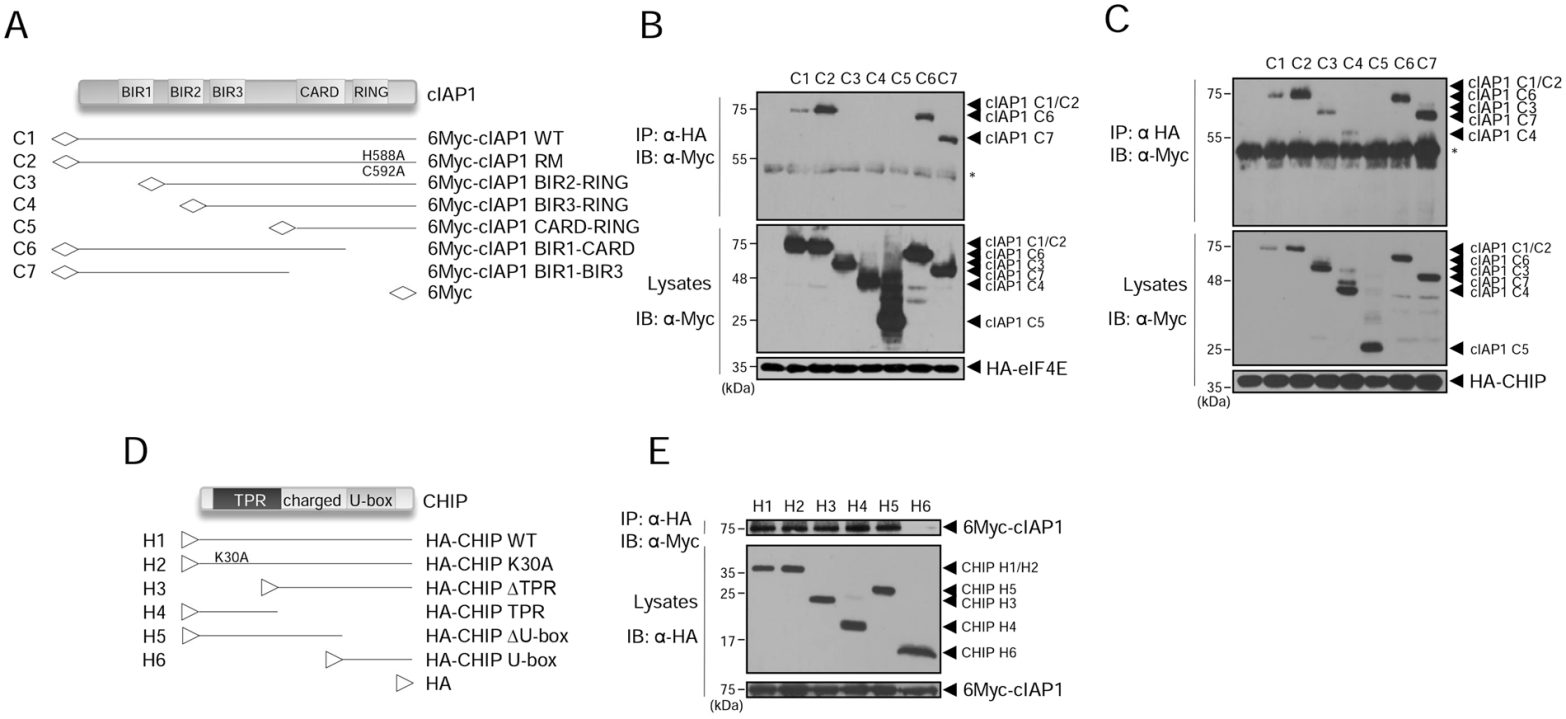
The BIR1 domain of cIAP1 is responsible for eIF4E binding while BIR3 is responsible for CHIP binding. (**A**) Schematic representation of the cIAP1 WT, RING domain mutant (H588A/C592A), and various truncation constructs used in the study. (**B**) HEK 293Tcells were co-transfected with the various 6Myc-cIAP1 constructs (**A**) and HA-eIF4E. After 24 h, WCL were co-immunoprecipitated with anti-HA antibody and analyzed by WB with anti-Myc antibody. *IgG heavy chain. (**C**) HEK 293 T cells were co-transfected with the various 6Myc-cIAP1 constructs (**A**) and HA-CHIP. After 24 h, WCL were co-immunoprecipitated with anti-HA antibody and analyzed by WB with anti-Myc antibody. *IgG heavy chain. (**D**) Schematic representation of the CHIP WT, K30A, and various truncation constructs. (**E**) HEK 293 T cells were co-transfected with 6Myc-cIAP1 and the various HA-CHIP constructs (**D**). After 24 h, WCL were co-immunoprecipitated with anti-HA antibody and analyzed by WB with anti-Myc antibody. WB images are cropped from the original blots shown in supplementary information.

To map the region of cIAP1 that interacts with eIF4E, we generated a series of truncation mutants of cIAP1 (Fig. 6A) and expressed them in HEK 293 T cells. The co-IP results showed that full-length cIAP1 constructs WT (C1) and RING mutant (C2) interacted with eIF4E. Truncated mutants containing the BIR1 domain, such as BIR1-CARD (C6) and BIR1-BIR3 (C7), interacted with eIF4E even more strongly (Fig. 6B). However, any cIAP1 mutants lacking BIR1, including BIR2-RING(C3), BIR3-RING (C4), and CARD-RING (C5), did not bind to eIF4E (Fig. 6B, C3–C5), indicating that the BIR1 domain of cIAP1 is necessary for the interaction with eIF4E. Next, to map the region of cIAP1 that interacts with CHIP, we co-transfected HEK 293 T cells with HA-CHIP and Myc-cIAP1 WT or one of the truncation mutants shown in Fig. 6A. Myc-cIAP1 WT and all mutants except Myc-cIAP1 CARD-RING (C5) bound to CHIP to some extent, indicating that BIR3 of cIAP1 is responsible for the interaction with CHIP (Fig. 6C). We also generated a series of truncation mutants of CHIP (Fig. 6D) and performed co-IPs with Myc-cIAP1 WT. The tetratricopeptide (TPR) domain of CHIP is known to mediate an interaction with Hsp70 and the U-box is the domain with which E2 interacts^34^. cIAP1 interacted with CHIP WT, the mutants containing the TPR domain, and K30A (H1–H3, H5). Interestingly, the mutant missing only the TPR domain (H4) also interacted with cIAP1, whereas the mutant lacking both the TPR and charged domains (H6) did not interact with cIAP1, indicating that cIAP1 interacts with CHIP via the TPR domain and the charged domain seems to be additionally required (Fig. 6E). In conclusion, the association between CHIP and eIF4E is mediated by the BIR3 domain of cIAP1 for CHIP binding and the BIR1 domain for eIF4E binding.

### The collaborative activity of cIAP1 and CHIP regulates eIF4E

**Figure 7:**
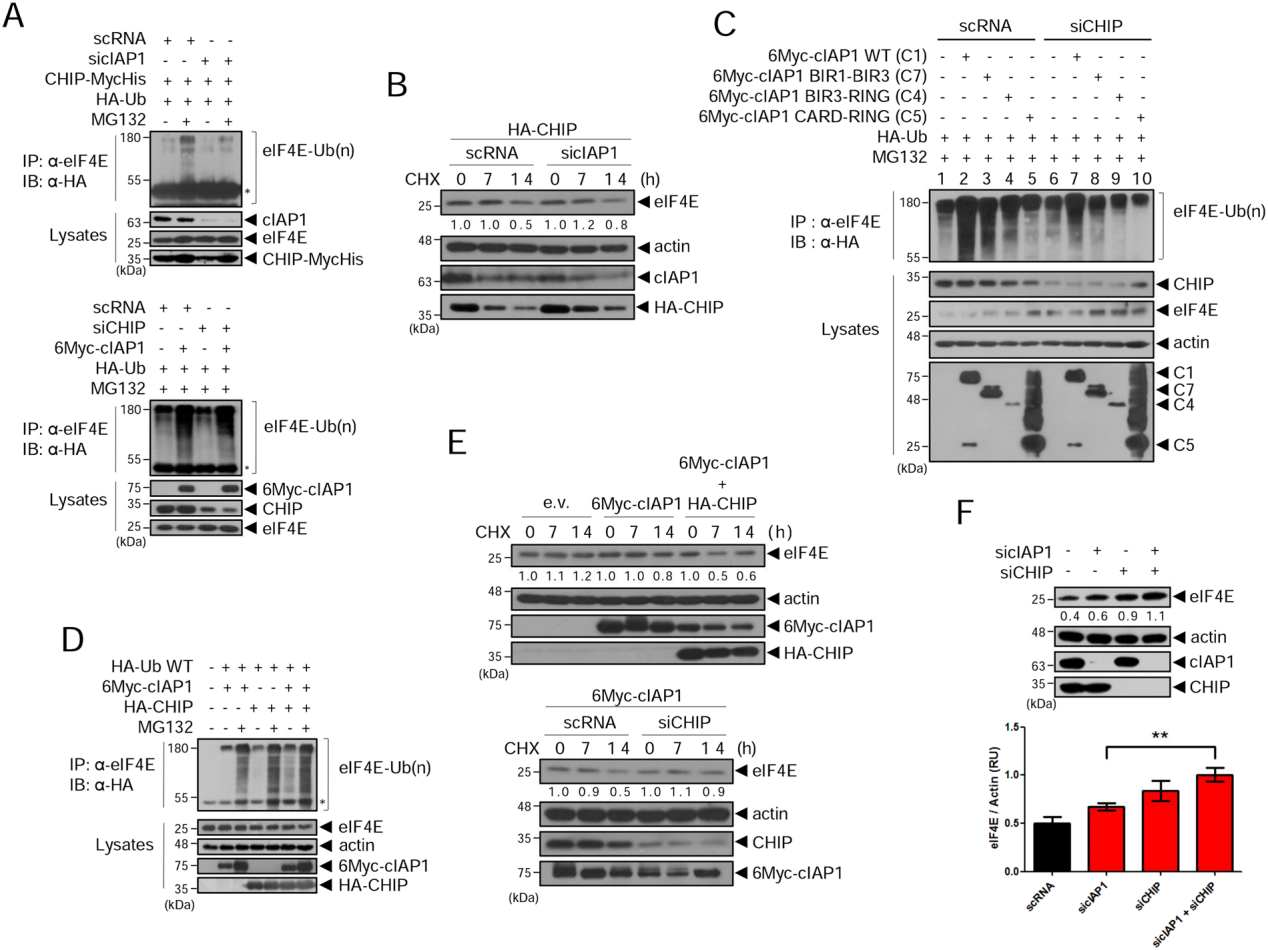
The collaborative activity of cIAP1 and CHIP regulates eIF4E. (**A**) HEK 293 T cells were transfected with sicIAP1 (top) or siCHIP (bottom) for 24 h, then co-transfected with HA-ubiquitin and CHIP-MycHis (top) or 6Myc-cIAP1 (bottom). After 24 h, cells were treated with 20 μM MG132 for 6 h, and WCL were co-immunoprecipitated with anti-eIF4E antibody, followed by WB with anti-HA antibody. *IgG heavy chain. (**B**) HeLa cells were transfected with scRNA or sicIAP1. After 24 h, cells were transfected with HA-CHIP for 24 h and half-life was measured at the indicated times. WCL were analyzed by WB with the indicated antibodies. The numbers indicate relative eIF4E expression to actin within the indicated condition. (**C**) HEK 293 T cells were transfected with scRNA or siCHIP. After 24 h, cells were co-transfected with various 6Myc-cIAP1 constructs (C1, C4, C5 and C7 in Fig. 6A) and HA-ubiquitin for 24 h. After 24 h, 20 μM of MG132 was treated for 6 h. Co-immunoprecipitations were performed using anti-eIF4E antibody and analyzed by WB with anti-HA antibody to reveal ubiquitinated eIF4E. (**D**) HEK 293 T cells were co-transfected with 6Myc-cIAP1, HA-CHIP, or both, and HA-ubiquitin. After 24 h, cells were treated with 20 μM MG132 for 6 h and WCL were co-immunoprecipitated with anti-eIF4E antibody, followed by WB with anti-HA antibody. *IgG heavy chain. (**E**) Measurement of eIF4E half-life in HeLa cells expressing 6Myc-cIAP1 alone or 6Myc-cIAP1 and HA-CHIP together (top), and in HeLa cells transfected with 6Myc-cIAP1 and scRNA or siCHIP (bottom). The numbers indicate relative eIF4E expression to actin within the indicated condition. (**F**) HeLa cells were depleted with either sicIAP1 (sicIAP1-1), siCHIP or both. After 48 h, WCL were analyzed by WB with the indicated antibodies (top). The numbers indicate expression of eIF4E relative to actin. Means ± SEM from three independent experiments, **p < 0.01, one-way ANOVA (bottom). WB images are cropped from the original blots shown in supplementary information.

Next, we investigated whether cIAP1 might function as a mediator in the regulation of eIF4E by CHIP. First, we examined eIF4E ubiquitination by CHIP when cIAP1 was depleted. Endogenous eIF4E was ubiquitinated by overexpressed CHIP in control cells, but this ubiquitination almost disappeared in cIAP1-depleted cells (Fig. 7A, top). On the contrary, eIF4E was well ubiquitinated by overexpressed cIAP1 in CHIP-depleted cells (Fig. 7A, bottom). Furthermore, the half-life of eIF4E was extended in cIAP1-depleted cells despite overexpression of CHIP (Fig. 7B). These results imply that cIAP1 has dual functions in eIF4E regulation: as an E3 ligase by itself, as well as a mediator between eIF4E and CHIP. To further investigate the mediator function of cIAP1 in eIF4E regulation by CHIP, we compared eIF4E ubiquitination by cIAP1 WT and by two types of mutants: one with the minimal regions for interaction with eIF4E and CHIP but no E3 ligase activity (C7: BIR1–BIR3), and those retaining ligase activity but unable to interact with eIF4E (C4: BIR3–RING) or with eIF4E and CHIP both (C5: CARD–RING). We observed considerable eIF4E ubiquitination in C7-expressing cells (lane 3) compared to that in cIAP1 WT-expressing cells (lane 2), but C4- or C5-expressing cells (lane 4, 5) showed only background levels of eIF4E ubiquitination (lanes 1 and 6) (Fig. 7C, top). When the same experiments were performed in CHIP-depleted cells, eIF4E ubiquitination was observed only in cIAP1 WT-expressing cells (Fig. 7C, top, lane 7) and the C7 (lane 8) and other mutants (lane 9, 10) showed only background levels (lane 1 or 6). These results are in accordance with the binding domain analysis (Fig. 6). Altogether, these data suggest that cIAP1 functions as a mediator of eIF4E regulation by CHIP and its RING activity might not be necessary for its mediator function.

We next sought to investigate whether eIF4E ubiquitination is enhanced by the collaborative activity of cIAP1 and CHIP, which we define as both the E3 ligase activity of cIAP1 alone and the E3 ligase activity of cIAP1/CHIP together. To this end, we assayed eIF4E ubiquitination, protein levels, and half-life in the presence of cIAP1, CHIP, or both. The strongest eIF4E ubiquitination was observed in cIAP1 and CHIP co-expressing cells, and consequently eIF4E protein levels in the cell lysate decreased the most (Fig. 7D) and the half-life of eIF4E protein was reduced (Fig. 7E) in these cells. We also compared the levels of eIF4E in cells depleted of cIAP1, CHIP, or both. As we expected, the steady-state levels of eIF4E were the most elevated in cells depleted of both proteins. (Figs 7F and S8). Altogether, our results demonstrate that cIAP1 both regulates eIF4E directly by itself as an E3 ligase and mediates the interaction of eIF4E with CHIP, and thereby the efficient regulation of eIF4E by CHIP. At the beginning of this study, we hypothesized that eIF4E might be regulated separately via two different E3 ligases. However, the results described lead us to conclude that eIF4E is efficiently regulated by the collaborative activity of cIAP1 and CHIP, which involves dual functions of cIAP1.

### The collaborative activity of cIAP1 and CHIP regulates eIF4E and cell growth of breast cancer cell lines

**Figure 8:**
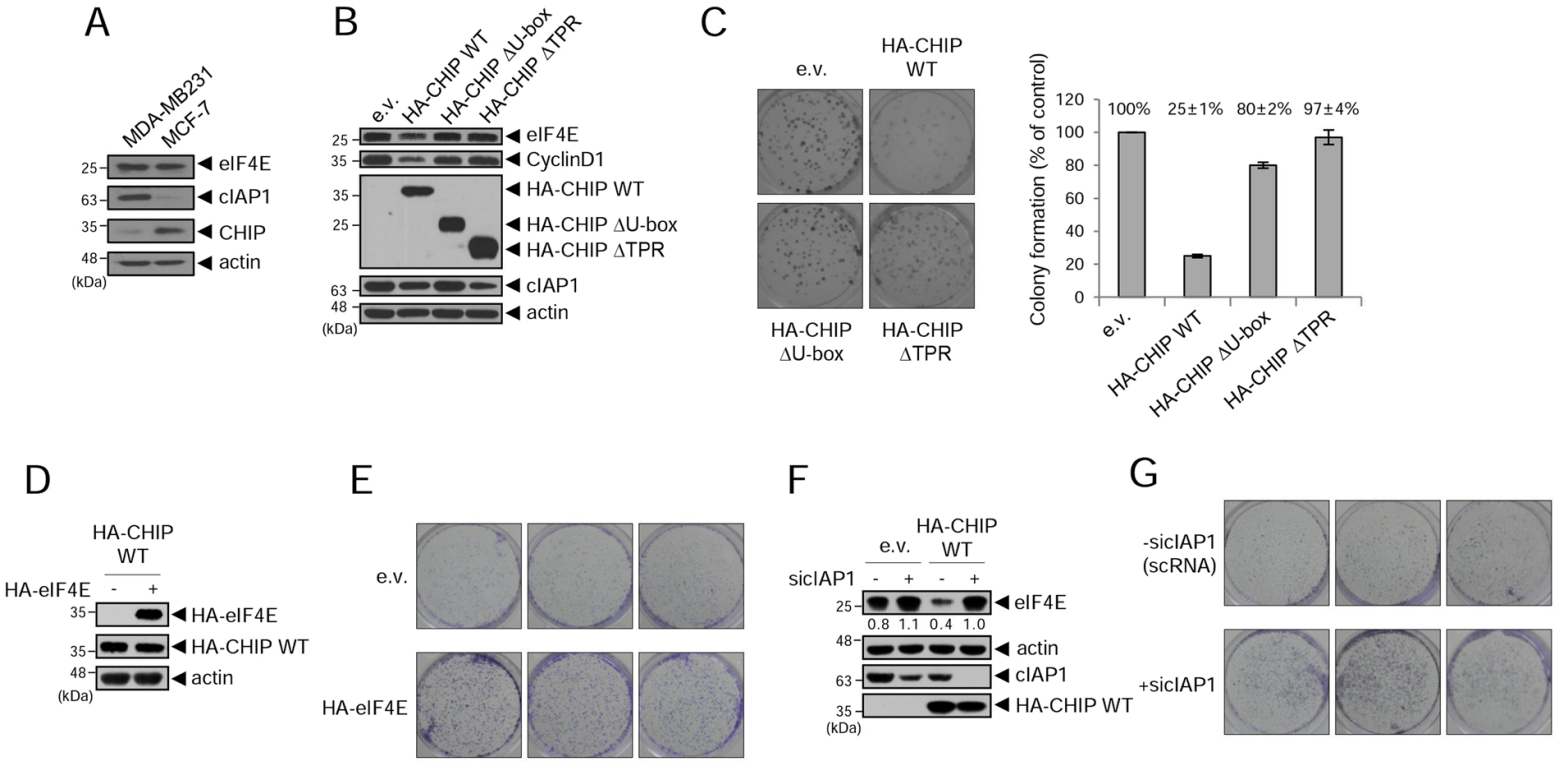
Depletion of cIAP1 reverses the reduced growth of CHIP WT-overexpressing MDA-MB231 cells. (**A**) The protein levels of endogenous eIF4E, cIAP1, and CHIP in MDA-MB231 and MCF7 cells. (**B**) MDA-MB231 cell lines stably expressing empty vector, HA-CHIP WT, HA-CHIP ∆U-box, and HA-CHIP ∆TPR were generated. WCL were analyzed by WB with the indicated antibodies. (**C**) The stable cell lines were grown in medium containing G418 for 2 weeks. Colonies were stained with crystal violet and photographed (left panel) and visible colonies were counted (right panel). Results are depicted as mean ± S.D. from three independent experiments. (**D** and **E**) MDA-MB231 cell lines stably expressing CHIP WT were transfected with HA-eIF4E. WCL were analyzed by WB with anti-HA antibody (**D**) and the colony formation assay was carried out for 2 weeks (**E**). (**F** and **G**) MDA-MB231 cell lines stably expressing empty vector or CHIP WT were transfected with scRNA or sicIAP1. WCL were analyzed by WB with indicated antibodies (**F**) and colonies were stained using crystal violet (**G**). The numbers indicate expression of eIF4E relative to actin. WB images are cropped from the original blots shown in supplementary information.

Overexpression of eIF4E is associated with aggressiveness, recurrence, and related death in breast cancer^13, 14^. Therefore, we investigated the physiological relevance of the collaborative regulation of eIF4E by cIAP1 and CHIP in breast cancer cell lines. We chose aggressive (MDA-MB231) and mild (MCF7) breast cancer cell lines and confirmed high expression of eIF4E in both (Fig. 8A), as previously reported^17^. Interestingly, we found the levels of cIAP1 and CHIP were inversely correlated in these cell lines (Fig. 8A). We postulated that low levels of cIAP1 (MCF7) or CHIP (MDA-MB231) may not be enough to generate collaborative activity for eIF4E regulation, leading to elevated eIF4E, and possibly to the tumorigenic phenotype. Therefore, we generated MDA-MB231 cell lines stably expressing HA-CHIP WT or the truncation mutants (HA-CHIP ∆U-box and HA-CHIP ∆TPR) and asked whether balanced expression of cIAP1 and CHIP proteins might generate collaborative eIF4E regulation activity, which would affect the tumorigenic phenotype. eIF4E was downregulated in HA-CHIP WT-expressing MDA-MB231 cells compared to control or truncation mutant-expressing cells, and the levels of cyclin D1, a well-known target of eIF4E, were also downregulated in proportion to eIF4E (Fig. 8B). Stable expression of CHIP WT reduced colony formation to about 20% of that in control cells, while the truncation mutant expression showed no difference compared to the control (Fig. 8C), suggesting that the reduction of eIF4E levels by CHIP WT expression might be linked to suppression of cell growth. When we increased the levels of eIF4E protein in CHIP WT-expressing MDA-MB231 cells by overexpression of HA-eIF4E (Fig. 8D), the colony formation recovered, indicating that the reduced colony formation was due to downregulation of eIF4E levels (Fig. 8E). Next, we depleted cIAP1 in either control or CHIP WT-expressing MDA-MB231 cells and observed the levels of eIF4E. The downregulation of eIF4E in CHIP WT-expressing cells was reversed by cIAP1 depletion (Fig. 8F), in accordance with the results shown in Fig. 7A,B, and F. The colony formation reduced by CHIP WT expression was also re-stimulated in cIAP1-depleted cells (Fig. 8G), implying that the elevated eIF4E was due to a lack of collaborative activity due to the absence of the dual functions of cIAP1 as an E3 ligase and a mediator. Altogether, these results indicate that the collaborative activity of cIAP1 and CHIP is crucial in determining the levels of eIF4E and the related tumorigenic phenotype in breast cancer cell lines.

### The RING activity of cIAP1 is not absolutely required for its mediator function

**Figure 9:**
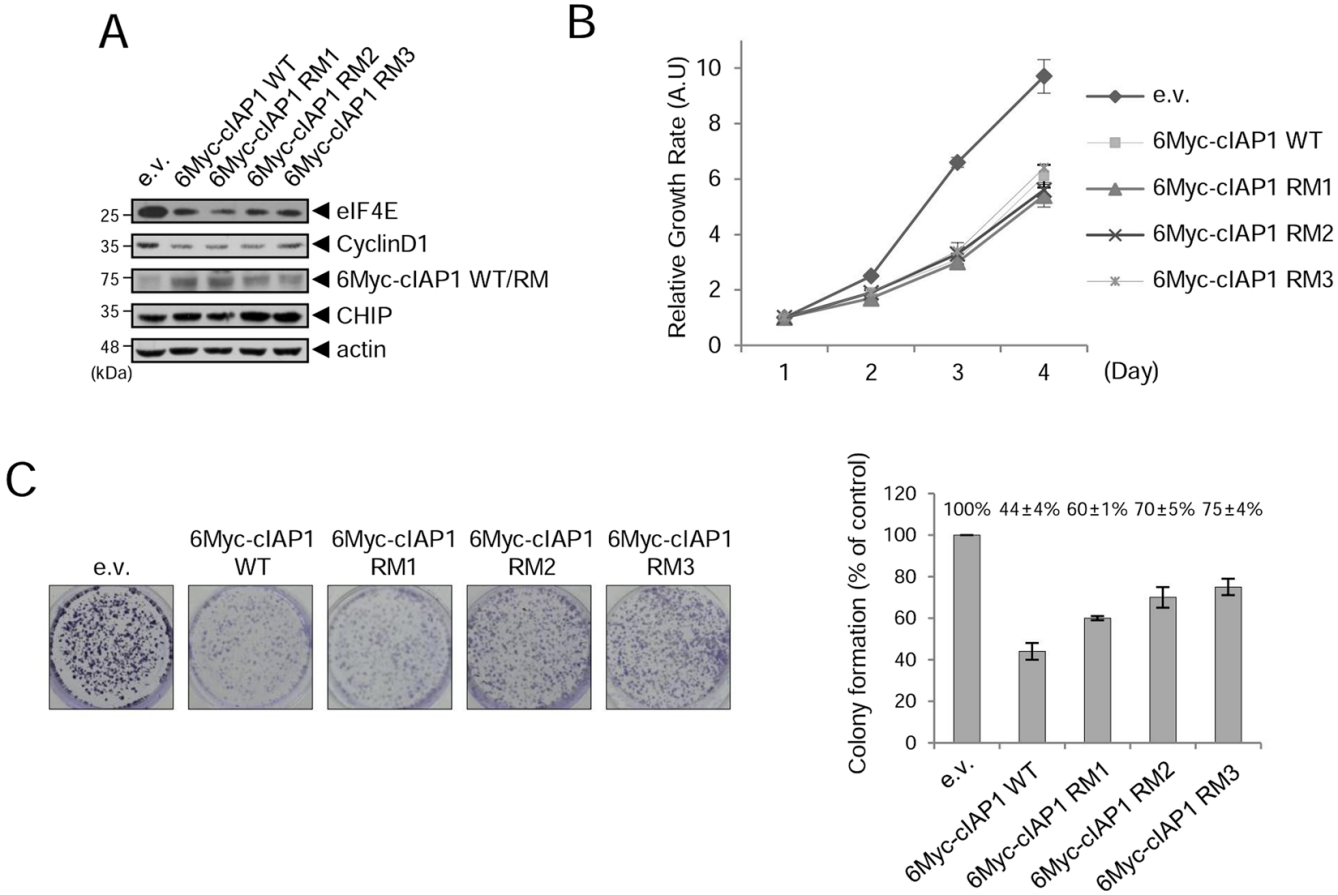
The RING activity of cIAP1 is not absolutely required for its mediator function. (**A**) MCF-7 cell lines stably expressing empty vector, 6Myc-cIAP1 WT, or 6Myc-cIAP1 RM were generated. WCL were analyzed by WB with indicated antibodies. (**B**) Cell proliferation was measured using the MTT assay for a total of 4 days in MCF-7 cell lines stably expressing indicated cIAP1 constructs. (**C**) Colony formation assay was carried out in medium containing G418 for 2 weeks. Colonies were stained with crystal violet and photographed (left panel) and visible colonies were counted (right panel). Results are depicted as mean ± S.D. from three independent experiments.

Next, we generated MCF7 cell lines stably expressing cIAP1 WT (6Myc-cIAP1 WT) or RING mutant (6Myc-cIAP1 RM) and investigated whether the levels of eIF4E were affected in these cells. eIF4E was downregulated in cIAP1 WT- and cIAP1 RM-expressing MCF7 cells compared to control cells (Fig. 9A). We used three cIAP1 RM-expressing cell lines (6Myc-cIAP1 RM1–3) which expressed different levels of cIAP1 RM. Interestingly, the levels of eIF4E were inversely correlated to the expression levels of cIAP1 RM protein, and subsequently the levels of cyclin D1 were proportional to eIF4E. Further, proliferation was suppressed in the cIAP1 WT-expressing MCF7 cells to about 50% of control (Fig. 9B) and cIAP1 RM expression also showed a similar reduction. The extent of colony formation was correlated with the levels of eIF4E in cIAP1 WT- or RM-expressing MCF7 cells regardless of RING activity (Fig. 9C). Altogether, these results suggest that the RING activity of cIAP1 is not absolutely required for its mediator function in the regulation of eIF4E and the related tumorigenic phenotype.

## Discussion

In the present study, we demonstrated that eIF4E was regulated by dual mechanisms that have in common the involvement of cIAP1. cIAP1 by itself is able to recognize and ubiquitinate eIF4E for proteasomal degradation, and cIAP1 also mediates the action of CHIP on eIF4E, together cooperatively enhancing the regulation of eIF4E.

Although cIAP1 is able to bind to caspases via its BIR domains, it is not a potent caspase inhibitor^35^. Rather, as an E3 ligase, cIAP1 inhibits cell death by ubiquitinating caspases^36^ or RIP1 kinase, preventing caspase 8 activation^6^. A role for cIAP1 has been demonstrated in a wide range of biological events beyond the regulation of cell death^3, 4, 37^. Our study provides the first evidence that cIAP1 functions as a mediator to support another E3 ligase for regulation of a specific protein. More interestingly, we found that cIAP1 mutants containing the BIR1–3 domains but with inactive or missing RING domains were able to support eIF4E regulation by CHIP. This indicates that E3 ligase activity may not be absolutely required to exert this mediator function, and the BIR1–3 domains of cIAP1 might be the minimal required region. Presently, it is unclear whether endogenous cIAP1 is processed to a fragment containing BIR1–3. However, we frequently observed processed cIAP1 fragments by WB (our unpublished data) and cIAP1 cleavage by caspases^38, 39^ and serine protease^40^ have been reported. Caspase 3 cleaved cIAP1 into two fragments; the C-terminal RING domain containing fragment had pro-apoptotic effects while the N-terminal BIR domain containing fragment was protective against cell death^39^. Together, these suggest that a cIAP1 fragment without the RING domain may exist and exert a mediator function.

As a cochaperone E3 ligase, CHIP has been known for regulating proteins presented by molecular chaperones. However, recent reports presented the possibility that CHIP might use alternative mechanisms to regulate specific protein substrates, as CHIP interacts with specific target proteins independent of molecular chaperones^20, 23, 41^, including some direct physical interactions^21, 22^. In the present study, we found that cIAP1 physically binds to CHIP via its BIR3 domain and to eIF4E via BIR1, which enables CHIP to regulate eIF4E without a chaperone. Therefore, cIAP1 is likely to replace the molecular chaperone in CHIP-driven eIF4E ubiquitination. We observed that other human IAPs are able to interact with CHIP (our unpublished data), suggesting a system of IAP/CHIP complexes might exert efficient substrate regulation. Indeed, XIAP has been shown to promote CHIP-driven ubiquitination of c-RAF, although Hsp90 was still required^37^. Therefore, the role of IAPs in CHIP-driven ubiquitination might vary depending on the target proteins. Why would cells use cIAP1 instead of molecular chaperones in CHIP-driven eIF4E regulation? CHIP has long been regarded as a quality-control E3 ligase because it regulates misfolded or abnormal proteins presented by molecular chaperones^16^. However, considering recent findings that CHIP is involved in the regulation of a number of specific target proteins, the existence of multiple mediators, including molecular chaperones and cIAP1, would provide a mechanism to regulate a wide variety of target proteins and thereby expand the scope of CHIP-driven ubiquitination. Several proteins have been reported to be regulated by either IAPs or CHIP^9, 42–44^. Therefore, it will be of great interest to revisit chaperone-independent target protein regulation by CHIP and ask whether cIAP1 (or other mediators) might replace the molecular chaperone in CHIP-driven target regulation in each particular context.

In the ubiquitin-proteasome system, although multiple E3 ligases may exist for a single substrate, each E3 ligase is usually used to ubiquitinate the substrate only in certain circumstances. However, reports have accumulated showing that E3 ligases can also collaborate and exert an advanced level of protein regulation to ensure proper degradation. For example, Mgt1 is regulated by cooperative activity of Ubr1, an E3 ligase of the N-end rule pathway, and Ufd4, an E3 ligase of the ubiquitin-fusion degradation pathway^45^. gp78 and TRIM25 together regulate AMF^46^, and gp78 cooperates with RMA1 in the ERAD pathway^47^. CHIP has been shown to cooperate with Parkin for PaeI-R regulation^30^, and with SCF^skp2^ for E47 regulation^31^. These examples all show that E3 ligase collaboration allows proper regulation through enhanced ubiquitination. Our results also demonstrate that cIAP1 alone is not enough to control the levels of eIF4E to prevent cancer cell growth and, therefore, cIAP1 and CHIP collaboration is required for proper control of eIF4E and prevention of tumorigenesis.

Presently, the precise role of CHIP in collaboration with other E3 ligases^30, 31^ has been described as that of an E4. An E4 is defined as an additional conjugation factor for efficient multi-ubiquitination together with E1–E3 enzymes^48^, and alternatively as a specialized E3 ligase that elongates the ubiquitin chain of a mono- or oligo-ubiquitinated substrate^49^. However, we hesitate to describe CHIP as an E4 in the regulation of eIF4E, because cIAP1 mutants without E3 ligase activity were able to support CHIP-driven ubiquitination in our experiments, and therefore prior ubiquitination by cIAP1 does not seem to be necessary. In this regard, we believe that the regulatory mechanism of eIF4E by cIAP1 and CHIP collaboration is probably different from that in the previous studies^27, 31^, and further analysis of eIF4E ubiquitination would provide better mechanistic insight into the collaborative activity of cIAP1 and CHIP.

Aberrant protein synthesis is a common characteristic found in cancers, and therefore the translation machinery is a reasonable target for the development of therapeutic agents. Indeed, eIF4E-specific antisense oligonucleotides (4E-ASOs) suppressed tumorigenic properties in vitro^50^ and *in vivo* ^51^, and phase I^52^ and II^53^ clinical trials of 4E-ASO are ongoing, indicating that reduction of eIF4E expression is a promising strategy.

High expression of IAPs is linked to tumorigenesis in various cancers^54^. So far, therapeutic strategies targeting IAPs aim to downregulate IAPs in cancer cells, and several IAP antagonist compounds (IACs) mimicking the natural IAP antagonist Smac have been developed, some of which are in clinical trials^54, 55^. However, our results indicate that reduced cIAP1 expression resulted in elevated eIF4E and the subsequent tumorigenic phenotype, implying that balancing the levels of cIAP1 and CHIP might be necessary to prevent tumorigenesis caused by eIF4E hyperactivity. In fact, most breast cancer cell lines are resistant to IACs^56^ and IACs sensitize only one-quarter of non-small-cell lung cancer cell lines^57^. Therefore, our data show that reducing cIAP1 is not the only direction for anti-cancer therapy, and therapies may need to be more careful in manipulating cIAP1 expression.

In breast cancer, CHIP levels are inversely correlated with malignancy and low CHIP expression results in elevated SRC-3, enhancing tumor growth and metastatic potentials^17^. Our data provide another mechanism by which CHIP plays a role in the suppression of breast cancer progression via regulation of eIF4E.

In summary, we identified a function of cIAP1 as a mediator of eIF4E regulation by CHIP, showing that collaboration of cIAP1 and CHIP efficiently controls eIF4E protein levels and prevents tumorigenesis. These findings provide insights into E3 collaboration mechanisms to improve specificity and efficiency of target degradation, suggesting that maintaining balanced levels of these E3 ligases might be a novel but plausible therapeutic strategy for the treatment of certain types of cancers.

## Methods

### Cell culture, transfection and RNA interference

HEK 293, HeLa, MDA-MB231, and MCF-7 cells were maintained in Dulbecco’s modified Eagle’s medium supplemented with 10% fetal bovine serum (WelGENE, Seoul, Korea). Stable breast cancer cell lines generated in this study were cultured in the presence of G418 (Duchefa, Haarlem, Netherlands). Transfection was performed using PEI (Sigma, St. Louis, MO), Lipofectamine 2000 (Invitrogen, Carlsbad, CA), X-tremeGENE Hp DNA transfection reagent (Roche, Mannheim, Germany) or Nucelofector Kit V (Lonza, Germany) according to the manufacturer’s recommendations. The sicIAP1, siCHIP, or control scRNA (Dhamacon, Chicago, IL) were transfected into HeLa cells using Lipofectamine 2000 (Invitrogen, Carlsbad, CA) according to the manufacturer’s instructions. WCL were analyzed 48 h after transfection by WB. The siRNA sequences included (sense strand, 5ʹ to 3ʹ): cIAP1-1, UCGCAAUGAUGAUGUCAAA and GAAUGAAAGGCCAAGAGUU (1:1 mixture of these two sicIAP1s used for cIAP1 depletion); cIAP1-2, UAUAGGACCUGGAGAUAGG; CHIP, CGCUGGUGGCCGUGUAUUA.

### Antibodies, co-immunoprecipitation, and immunostaining

Anti-cIAP1 (R&D Systems, Minneapolis, MN), anti-eIF4E (BD Biosciences, Franklin Lakes, NJ), anti-Myc, anti- His (Millipore, Billerica, MA), anti-HA (Covance, Emeryville, CA), anti-GST, anti-CyclinD1, anti-Hsp70 (Santa Cruz Biotechnology, Inc. Dallas, Texas), anti-β-actin (Bethyl, Montgomery, TX), and anti-CHIP (Youngin Frontier, Seoul, Korea) antibodies were used for WB or co-IP.

Co-IP experiments were performed as follows unless otherwise noted. Cells transfected with the indicated plasmids were lysed in lysis buffer (50 mM Tris pH 8.0, 150 mM NaCl, 1 mM EDTA, 1% Triton X-100, 10% glycerol, protease inhibitor cocktail). WCLs were mixed with the appropriate antibodies for 2 h at 4 °C. Immunocomplexes were incubated for 2 h with protein-A sepharose (Sigma) and the resin was washed three times with washing buffer (20 mM Tris pH 8.0, 150 mM NaCl, 1 mM EDTA, 0.1% Triton X-100, protease inhibitor cocktail). Samples were subjected to SDS-PAGE and analyzed by WB.

For general immunostaining, cells grown on 12-mm diameter coverslips were fixed in 3.7% paraformaldehyde in phosphate-buffered saline (PBS) for 20 minutes and permeabilized using 0.2% Triton X-100 in PBS for 15 min. Cells were blocked in 2% bovine serum albumin in PBS for 1 h and incubated with the indicated primary antibody overnight at 4 °C. After washing with PBS, cells were incubated with mouse Alexa Fluor 488-conjugated or rabbit Alexa Fluor 568-conjugated secondary antibody (Invitrogen). After washing, cells were counterstained with DAPI and mounted (Vector Laboratories, Inc. Burlingame, CA.), and visualized using a LSM 510 Meta confocal microscope (Carl Zeiss, Inc. Germany).

### Biomolecular fluorescence complementation assay

For the BiFC assay, cIAP1 and CHIP were subcloned into the pBiFC-VN 173 and pBi FC-VC 155 (a kind gift from Dr. Lee K. H., Chosun Univ.) vectors, respectively. HeLa cells were co-transfected with cIAP1-VN and CHIP-VC. After 24 hours, the cells were washed with PBS, fixed in 3.7% paraformaldehyde, and again washed twice with PBS. Cells were stained with DAPI and mounted on glass slides. Imaging and analysis were conducted according to previously described protocols^23^.

### GST pull-down assay

GST-cIAP1, GST-CHIP (WT, K30A), eIF4E-6His, and CHIP-6His (WT, K30A) were generated by cloning into pGEX4T-1 and pET 23a vectors, and recombinant proteins were expressed and purified in bacteria according to manufacturer’s instructions. Glutathione beads incubated with GST, GST-cIAP1, or GST-cIAP1 were incubated with eIF4E-6His or CHIP-6His in lysis buffer (100 mM Tris pH 8.0, 100 mM NaCl, 2 mM EDTA, 5% glycerol) for 2 h at 4 °C with rotation. The glutathione beads were washed three times with 1 ml of lysis buffer. Precipitated proteins were eluted by adding 1× SDS sample buffer and analyzed by WB with anti-His antibody.

### In vitro and in vivo ubiquitination assays

For the *in vitro* ubiquitination assay, purified eIF4E-6His was added to a reaction mixture consisting of 9 µg purified Ubiquitin-6His, 0.5 µg E1 enzyme (BostonBiochem, Cambridge, MA), and 10 µg purified E2 enzyme (GST-Ubc5a), in the presence or absence of purified GST-cIAP1 or GST-CHIP as an E3 ligase. All reactions were incubated at 37 °C, stopped by adding 1× SDS sample buffer, and analyzed by WB with the indicated antibodies. For the *in vivo* ubiquitination assay, cells were transfected with HA-tagged ubiquitin (HA-Ub) and the indicated plasmids. Cells were treated 24 h after transfection with 20 µM MG132 for 6 h, then lysed in lysis buffer (50 mM Tris pH 8.0, 150 mM NaCl, 1 mM EDTA, 1% Triton X-100, 10% glycerol, protease inhibitor cocktail). Co-IPs were performed using the appropriate antibody and analyzed by WB.

### Stable cell lines and colony formation assay

For establishment of stable cells lines, MDA-MB231 or MCF7 cells were transfected with CHIP WT and the various truncation mutants shown in Fig. 6, Myc-cIAP1 WT, or Myc-cIAP1 RM constructs using X-tremeGENE Hp DNA transfection reagent (Roche). The cells were selected by 0.7–1.2 µg/ml G418. Culture media containing G418 were replenished every three days. After three weeks of selection, colonies were picked, expanded, and the overexpression of CHIP or cIAP1 proteins was confirmed by WB using anti-HA or anti-Myc antibodies. For the colony formation assay, MDA-MB231 (2.5 × 10^3^) or MCF7 (5 × 10^3^) cells were plated in in a 6-well plate. Cells were maintained in the presence of G418 (0.7 µg/ml) for 2 weeks. The colonies formed from each cell were fixed with 4% paraformaldehyde for 30 min and then with 0.2% crystal violet in 10% ethanol for 40 min, and then colonies were counted. Each assay was performed in triplicate.

### Cell proliferation analysis

MCF-7 cell lines stably expressing Myc-cIAP1 WT or Myc-cIAP1 RM were seeded in 96-well plates (5 × 10^3^ per well). Cell proliferation was measured using the EZ-Cytox Cell viability assay kit (Dogen bio, Seoul, Korea) every day for a total of four days. The absorbance was measured at 450 nm using Multi-Mode microplate readers (BioTek, Winooski, VT). Each sample was assayed at least in triplicate.

## Electronic supplementary material

supplementary information
