# Supplementary material for: A novel function of cIAP1 as a mediator of CHIP-driven eIF4E regulation
Source: Sci Rep. 2017 Aug 29;7:9816. doi: 10.1038/s41598-017-10358-2 (PMC5575267; doi:10.1038/s41598-017-10358-2)
Supplement: Supplementary file 1 — supplementary information [file 41598_2017_10358_MOESM1_ESM.pdf]

Supplementary Information

A novel function of cIAP1 as a mediator of CHIP-driven eIF4E regulation

Tae Woong Seo[1b], Ji Sun Lee[1ab], Ye Na Choi[b], Dar Heum Jeong[a], Sun Kyung Lee[a], Soon Ji Yoo[a,b]*
[a]*Department of Life and Nanopharmaceutical Sciences and* [b]*Department of Biology, Kyung Hee University, Seoul 02447, Korea*

* To whom correspondence should be addressed.
Address: Department of Life and Nanopharmaceutical Sciences and Department of Biology, Kyung Hee University, 1, Hoegi-dong, Dondaemoon-gu, Seoul 130-701, Korea
Tel: 82-2-961-0978; Fax: 82-2-961-0244
E-mail address: yoosoonji@khu.ac.kr

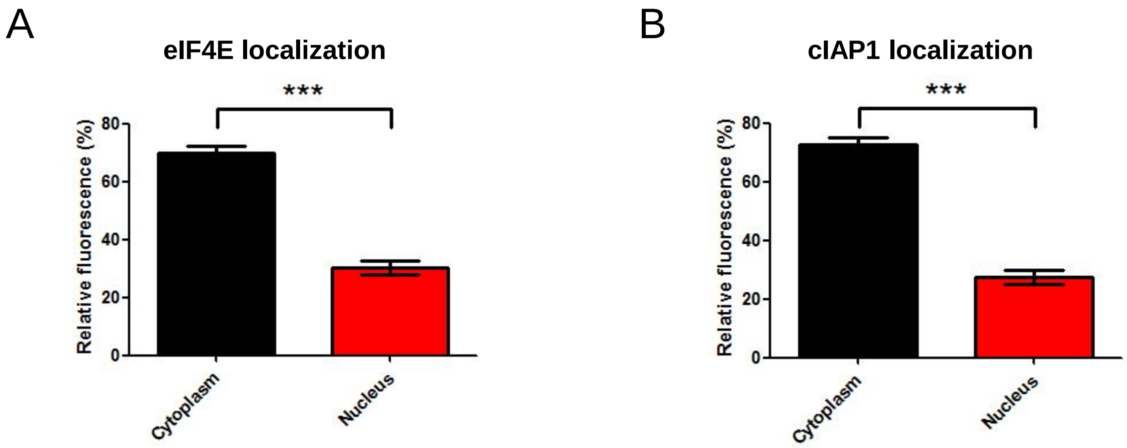

Figure S1. eIF4E and cIAP1 were mainly localized in the cytoplasm. Quantification of immunostained eIF4E (A) and cIAP1 (B) area per cell in Hela cells using the Image J program. Values represent mean ± s.e.m. from triplicate independent experiments (***$p < 0.0001$, unpaired t-test).

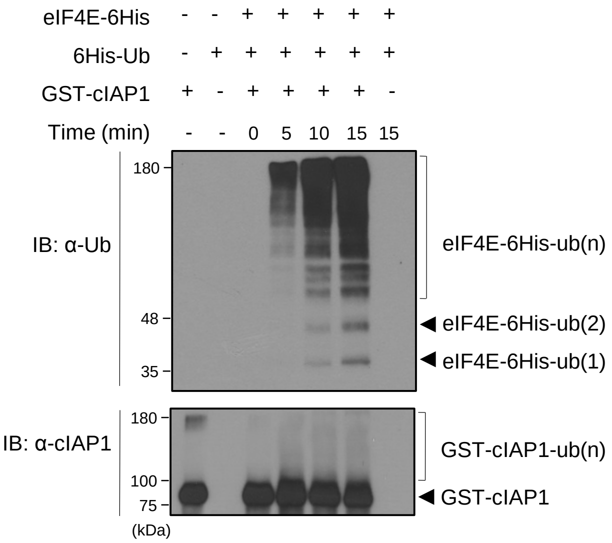

Figure S2. Purified eIF4E-6His and GST-cIAP1 proteins were subjected to the in vitro ubiquitination assay. The experimental conditions were same as Fig. 2B except for shorter incubation. After incubation, samples were analyzed by WB with anti-Ub (Top) or anti-cIAP1 antibody (bottom).

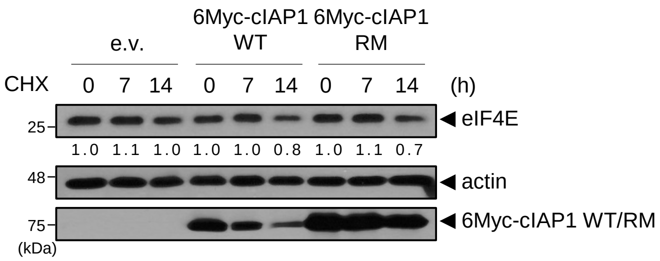

Figure S3. RING domain of cIAP1 is dispensable for CHIP-driven eIF4E regulation. HeLa cells were transfected with 6Myc-cIAP1 WT or Ring mutant (RM), or empty vector (e.v.). After 48 h, cells were treated with 200 μg/ml cycloheximide (CHX) and harvested at the indicated times. WCL were analyzed by WB with anti-eIF4E or anti-Myc antibodies. The numbers indicate relative eIF4E expression to actin within the indicated vector-expressing cells.

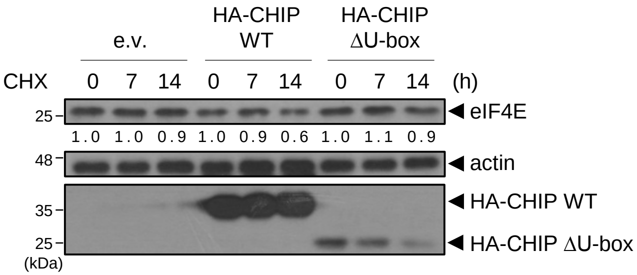

Figure S4. Comparison of eIF4E half-life on expression of HA-CHIP WT or HA-CHIP ΔU-box expression. HeLa cells were transfected with HA-CHIP WT, HA-CHIP ΔU-box, or empty vector (e.v.). After 48 h, cells were treated with 200 μg/ml cycloheximide (CHX) and harvested at the indicated times. WCL were analyzed by WB with anti-eIF4E or anti-HA antibodies. The numbers indicate relative eIF4E expression to actin within the indicated vector-expressing cells.

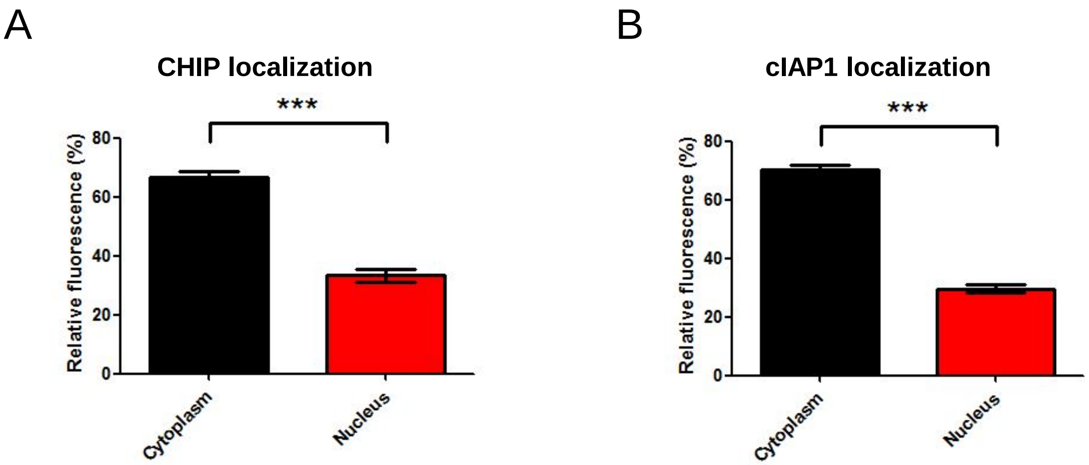

Figure S5. CHIP and cIAP1 were mainly localized in the cytoplasm. Quantification of immunostained CHIP (A) and cIAP1 (B) area per cell in Hela cells using the Image J program. Values represent mean ± s.e.m. from triplicate independent experiments (***$p < 0.0001$, unpaired t-test).

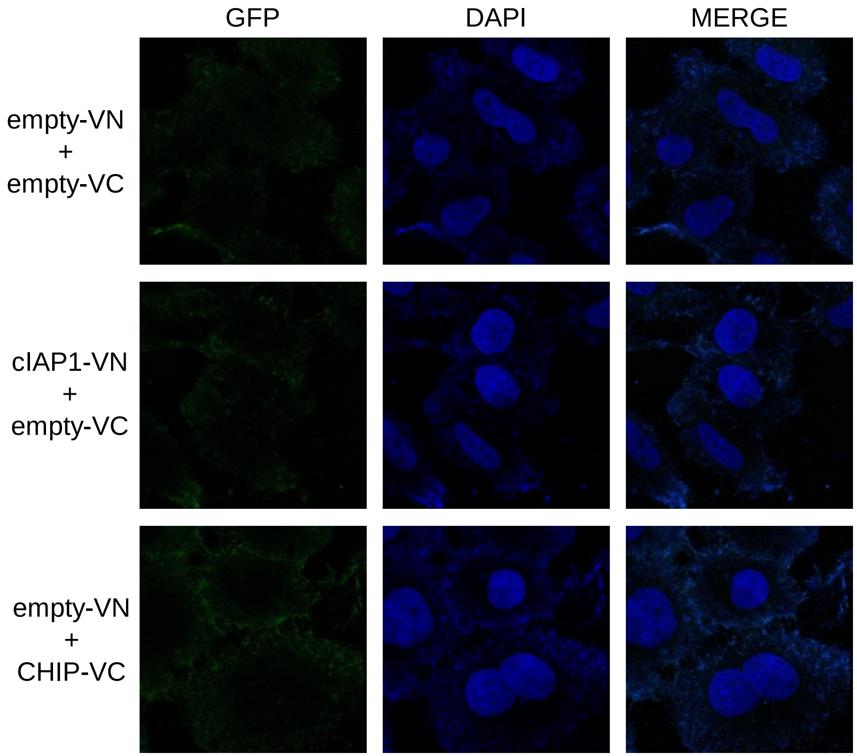

Figure S6. Images of control vector expression in BiFC assay shown in Figure 4D. Empty-VC and empty-VN vectors were used as control vectors in combination with either cIAP1-VN or CHIP-VC. The experimental conditions were same as Fig. 4D.

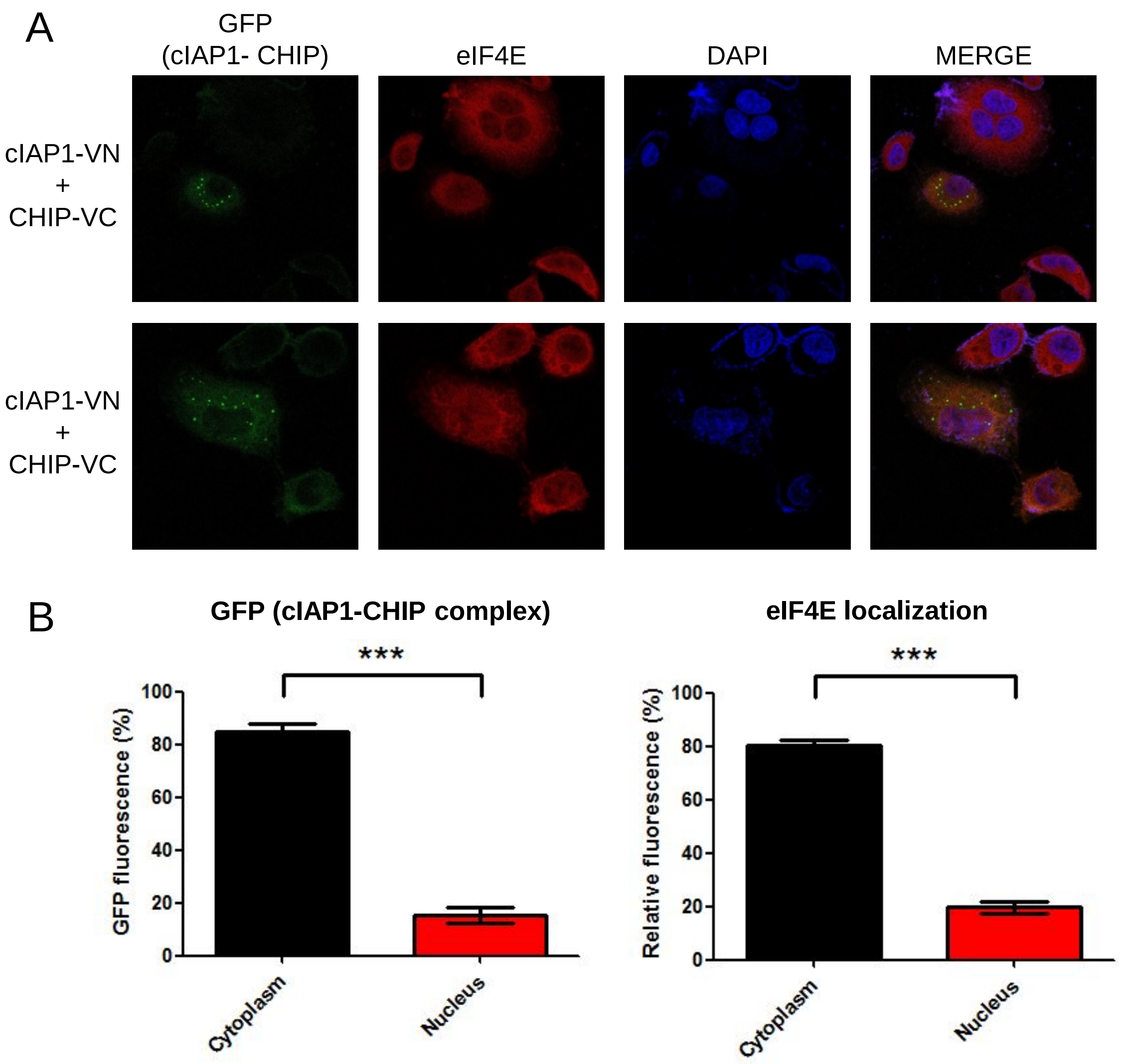

Figure S7. cIAP1 and CHIP were mainly co-localized with eIF4E in the cytoplasm. The experimental conditions for Bi-FC assay were same as Fig. 5D (A). Quantification of GFP fluorescence and eIF4E area per cell in Hela cells using the Image J program (B). Values represent mean ± s.e.m. from triplicate independent experiments (**p < 0.0001, unpaired t-test).

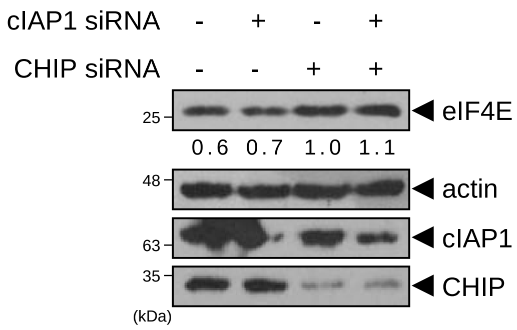

Figure S8. The collaborative activity of cIAP1 and CHIP regulates eIF4E. The experimental conditions were same as Fig. 7F, except for using sicIAP1-2 instead of sicIAP1-1. The numbers indicate expression of eIF4E relative to actin.

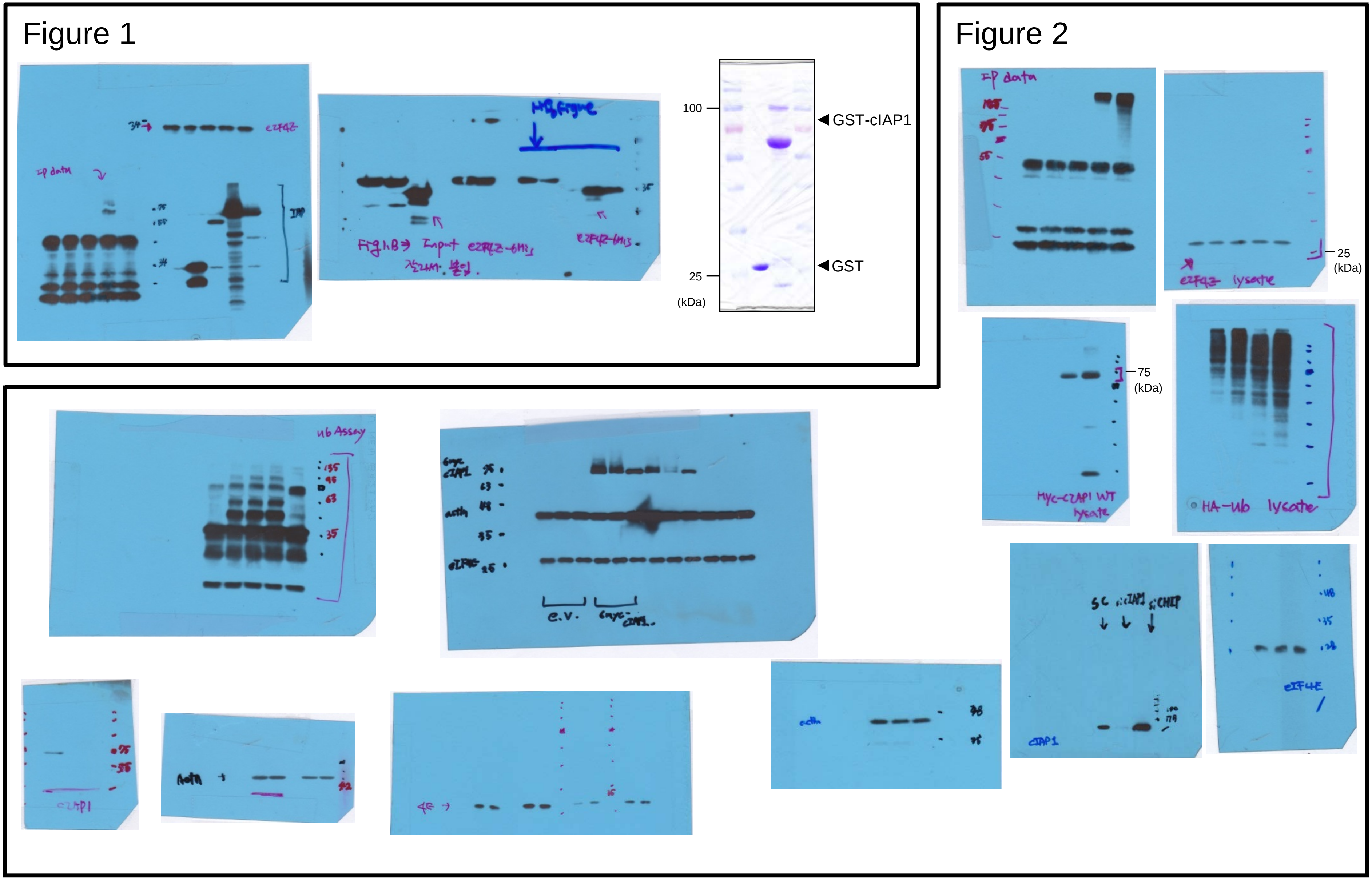

Figure S9. Original uncropped Western Blotting reported in Fig. 1 and 2.

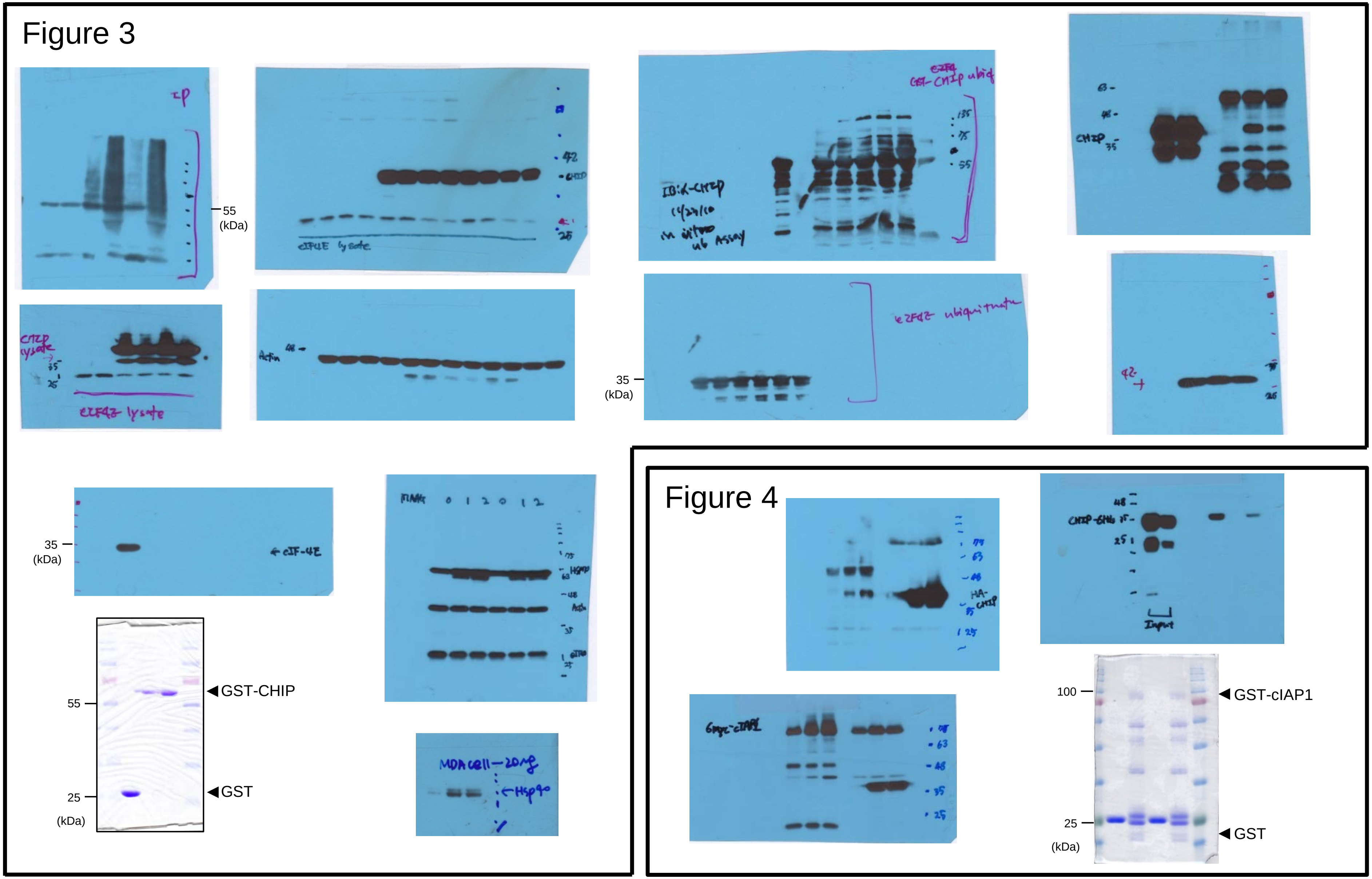

Figure S10. Original uncropped Western Blotting reported in Fig. 3 and 4.

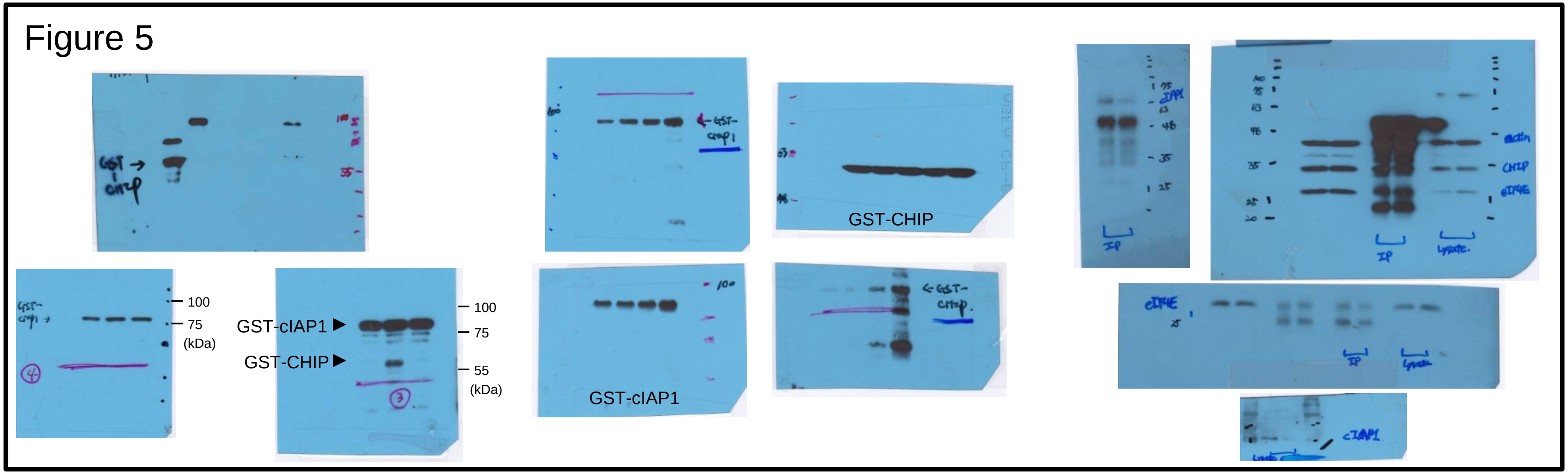

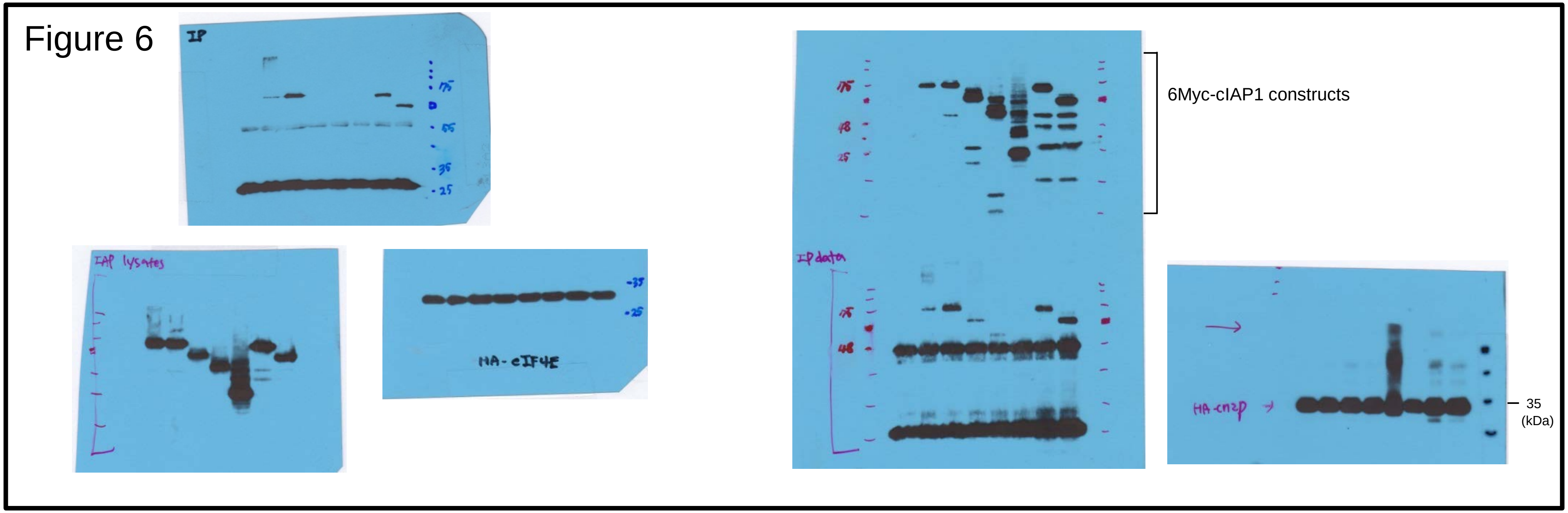

Figure S11. Original uncropped Western Blotting reported in Fig. 5 and 6.

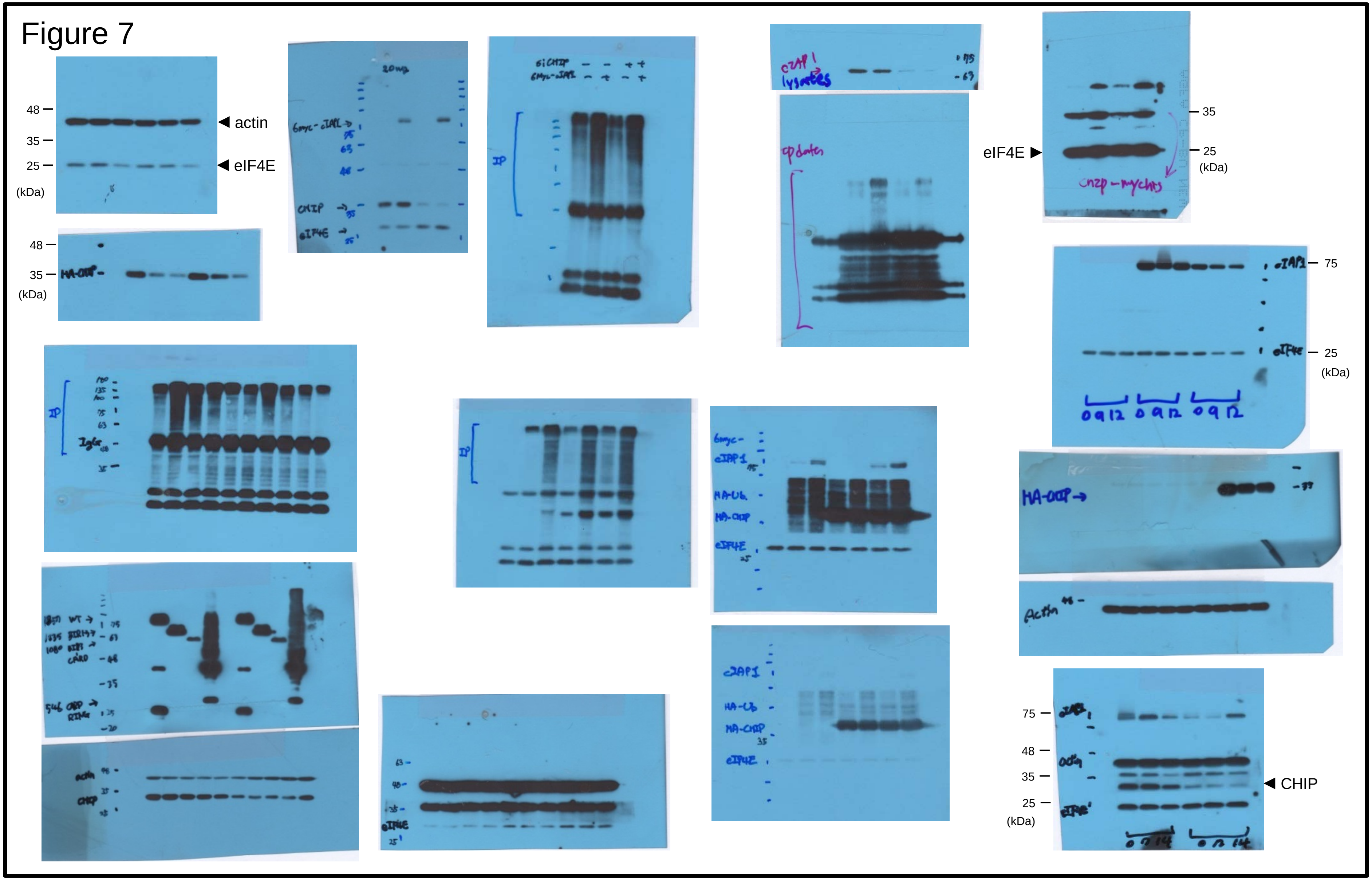

Figure S12. Original uncropped Western Blotting reported in Fig. 7.

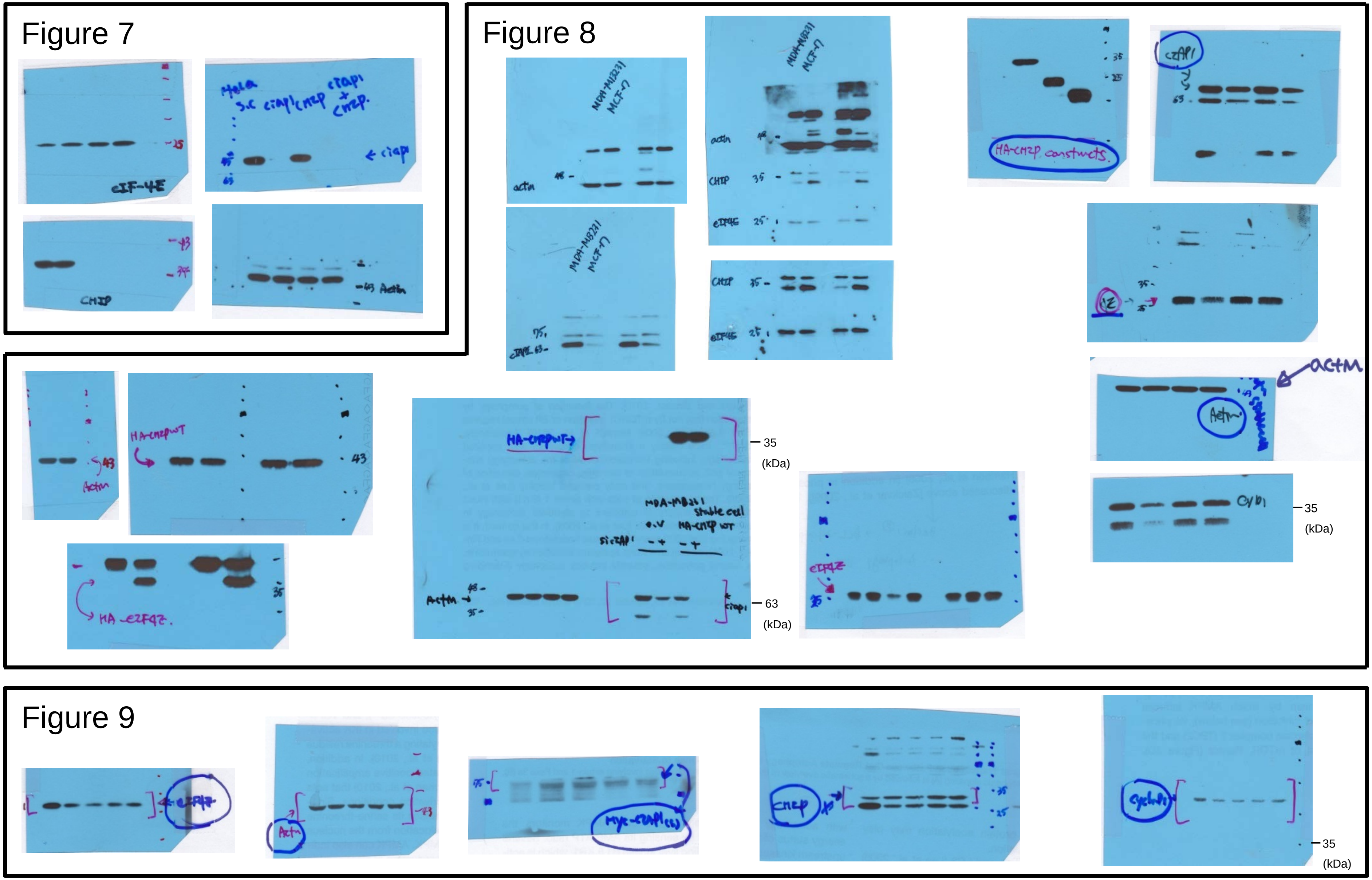

Figure S13. Original uncropped Western Blotting reported in Fig. 7, 8 and 9.
